# Reduced NEXI protocol for the quantification of human gray matter microstructure on the Connectome 2.0 scanner

**DOI:** 10.1162/IMAG.a.1380

**Published:** 2026-10-09

**Authors:** Quentin Uhl, Tommaso Pavan, Julianna Gerold, Kwok-Shing Chan, Yohan Jun, Shohei Fujita, Aneri Bhatt, Yixin Ma, Qiaochu Wang, Hong-Hsi Lee, Susie Y. Huang, Berkin Bilgic, Ileana Jelescu

**Affiliations:** Department of Radiology, Lausanne University Hospital (CHUV) and University of Lausanne, Lausanne, Switzerland; Department of Radiology, Massachusetts General Hospital, Athinoula A. Martinos Center for Biomedical Imaging, Boston, MA, United States

**Keywords:** diffusion MRI, gray matter microstructure, NEXI, protocol optimization, explainable AI, clinical translation

## Abstract

Biophysical diffusion MRI models such as Neurite Exchange Imaging (NEXI) are essential for probing gray matter microstructure, estimating compartment diffusivities (*D_i_*, *D_e_*), neurite fraction (*f*), and exchange time (*t_ex_*). However, NEXI multi-shell, multi-diffusion-time requirements lead to prohibitively long acquisitions. Leveraging the Connectome 2.0 ultra-high gradient scanner, we developed a time-efficient protocol using an Explainable AI (XAI) framework. Combining XGBoost, SHAP, and Recursive Feature Elimination trained on synthetic signals, XAI identified an optimal 8-feature subset, halving scan time from 27 to 14 minutes. Validated in vivo in 7 healthy participants, the XAI protocol was benchmarked against the full 15-feature acquisition, a Cramér-Rao Lower Bound (CRLB) theoretical optimum, and 2 expert-designed heuristics (“Mid-Range” and “Corner”). The XAI protocol robustly reproduced parameter estimates and maintained full protocol test–retest reproducibility. Remarkably, the data-driven XAI selection converged to the theoretical CRLB optimum. This validates XAI mathematical optimality while highlighting its primary advantage: achieving gold-standard optimization without requiring complex analytical Jacobians, making it effortlessly adaptable to numerically defined models or complex noise distributions where CRLB becomes intractable. Furthermore, XAI demonstrated superior in vivo robustness compared with heuristics: “Mid-Range” sampling yielded biased *t_ex_* estimates due to insufficient temporal diversity, while “Corner” sampling yielded unstable *D_i_* estimates (fivefold higher coefficient of variation) due to noise sensitivity. Ultimately, this robust 14-minute NEXI protocol accelerates exchange-sensitive microstructural mapping, establishing a generalizable, model-agnostic optimization framework adaptable to future ultra-high gradient systems (e.g., Siemens Cima.X or GE MAGNUS) and existing clinical scanners.

## 1 Introduction

Mapping cortical microstructure is a growing priority in neuroscience and clinical imaging, particularly for detecting early changes in neurodegenerative diseases such as Alzheimer’s disease, Parkinson’s disease, and multiple sclerosis, or in neuropsychiatric disorders such as schizophrenia (Atkinson-Clement et al., 2017; Dong et al., 2020; Spotorno et al., 2023). These pathologies are often marked by subtle alterations in gray matter cytoarchitecture, such as neurite beading, reduced spine or dendritic density, synaptic loss, and changes in membrane permeability, which precede detectable volumetric atrophy. Advanced diffusion MRI (dMRI) techniques now enable indirect but noninvasive quantification of these microstructural processes by modeling the mean squared displacement of water molecules in tissue (Basser et al., 1994; Le Bihan et al., 1991).

Biophysical dMRI models have extended the utility of diffusion imaging beyond empirical metrics to estimate biologically relevant parameters such as neurite density, intra- and extra-cellular diffusivities, exchange rates, and soma size (Alexander et al., 2019; Jelescu et al., 2020; Novikov et al., 2019; Stanisz et al., 1997).

However, the assumptions of the early models, largely developed for the aligned axonal structures of white matter, are challenged by the complex cytoarchitecture of cortical gray matter. Several model extensions have emerged to account for these effects, including SANDI (Palombo et al., 2020, 2018), which incorporates a spherical soma compartment, and NEXI (Jelescu et al., 2022), which models exchange between intra- and extra-neurite spaces. A parallel formulation of the same model, SMEX (Olesen et al., 2022), accounts also for finite gradient durations by solving the exchange equations numerically, which becomes essential when the narrow pulse approximation (NPA) breaks down, such as on standard clinical scanners with weaker gradients.

NEXI is particularly appealing for gray matter applications: by explicitly modeling exchange, it estimates an apparent exchange time *t_ex_*, alongside intra-neurite diffusivity *D_i_*, extra-neurite diffusivity *D_e_*, and the intra-neurite volume fraction *f*. Unlike SANDI, NEXI does not explicitly model the soma contributions, instead absorbing them into the extra-neurite compartment, a choice supported by evidence that exchange dominates signal evolution at standard diffusion times (>20 ms) (Jelescu et al., 2022; Lee et al., 2020; Olesen et al., 2022). Models such as SANDIX (Olesen et al., 2022), which incorporate both exchange and spherical restriction, offer a more comprehensive microstructural picture, but this richness comes at the cost of increased model complexity. Estimating a large number of parameters simultaneously can lead to unstable fits and unreliable estimates (Olesen et al., 2022).

Recent studies on 3 Tesla Connectome 1.0 and clinical-grade scanners have shown that NEXI and SMEX can be implemented in humans, and that the estimated exchange time correlates strongly with myelin-sensitive imaging metrics such as the myelin water fraction (MWF) (Uhl et al., 2024c, 2025). These findings establish *t_ex_* as a potential biomarker of membrane permeability and myelination, with spatial maps revealing biologically plausible patterns across the cortex. However, the underlying acquisition protocols are lengthy (typically > 30 minutes) as they require extensive sampling across both gradient amplitude and diffusion time dimensions (i.e., the acquisition of multi-b, multi-t data). The long acquisition time limits the scalability of NEXI in clinical and research settings where scan time, subject motion, and compliance are critical constraints (Illán-Gala et al., 2022; Spotorno et al., 2023).

The introduction of the Connectome 2.0 scanner (Huang et al., 2021; Ramos-Llordén et al., 2025) offers new opportunities for advanced dMRI protocol design. With 500 mT/m gradients and 600 T/m/s slew rates, it enables high b-values and short diffusion times (achieved with short gradient pulse separations, Δ), essential for probing fast exchange and structural disorder (Chan et al., 2025). Furthermore, the enhanced hardware performance provides a robust benchmark for validating estimations from clinical scanners with lower gradient strengths. On this platform, the standard NEXI model becomes more viable, as gradient pulse durations (δ = 5 ms) approach the idealized NPA, reducing the need for pulse-width corrections (Chan et al., 2025). Yet, even on Connectome 2.0, total acquisition time remains a bottleneck. Reducing the number of (*b*, Δ) combinations while preserving parameter accuracy is, therefore, essential for clinical translation.

Historically, the design of dMRI acquisition schemes has relied on “top–down” analytical frameworks aimed at maximizing the sensitivity of the signal to specific physical phenomena. Earlier efforts focused on the optimization of Diffusion Tensor Imaging (DTI) by determining the minimum number of gradient directions and optimal b-values required for robust anisotropy measurements (Jones et al., 1999). This matured into a generalized framework for experimental design based on the Fisher Information Matrix (FIM) and the Cramér-Rao Lower Bound (CRLB), which seeks to minimize parameter estimation variance (Alexander, 2008; Poot et al., 2010). Such model-based approaches have been successfully applied to white matter models (Coelho et al., 2022) and more recently extended to multidimensional dMRI encoding (Bocquillon et al., 2025), where dictionary-based greedy algorithms or CRLB-informed selection are used to navigate the vast parameter space of b-tensor shapes.

However, strictly relying on CRLB-based analytical optimization presents significant practical limitations and is not easily generalizable. Formulating the required analytical Jacobians is mathematically cumbersome or even intractable for advanced models accounting for complex effects, such as inter-compartmental exchange or restricted diffusion in non-idealized geometries. Furthermore, highly non-linear models such as NEXI or SMEX can be extremely sensitive to these Jacobian derivations, and the standard theoretical CRLB framework struggles to easily incorporate complex, non-Gaussian noise distributions (Karlsen et al., 1999) or hard physical boundary constraints. These mathematical bottlenecks have motivated the emergence of “bottom–-up” data-driven strategies that identify the most informative measurements without prior assumptions about the underlying distribution (Grussu et al., 2021). Recent advances in physics-informed machine learning have demonstrated that neural networks, such as Concrete Autoencoders, can be used to perform differentiable feature selection, effectively pruning comprehensive datasets to find optimal measurement subsets (Balın et al., 2019; Planchuelo-Gómez et al., 2024). While these methods excel at reconstructing the full signal from sparse samples, their primary focus is often signal prediction rather than the direct, interpretable quantification of biophysical parameters.

Despite these advances, a gap remains between purely theoretical CRLB optimization, which is locally defined and sensitive to the choice of a ground-truth “anchor” point, and deep-learning-based selection, which often operates as a black “box.” Explainable AI (XAI) offers a bridge by providing a statistically rigorous yet interpretable ranking of features based on their contribution to the final estimate. By leveraging global sensitivity analysis via SHAP values, XAI-driven optimization captures the non-linear degeneracies inherent in models such as NEXI while maintaining a clear link to the physical acquisition dimensions (*b*, Δ). This framework allows for a direct comparison with both heuristic expert designs and classical A-optimality, ensuring that the resulting protocol is not only efficient but also physically grounded and clinically reproducible.

To bridge this gap, we implement an XAI-oriented protocol reduction framework built around three key components: gradient-boosted decision trees (XGBoost) (Chen & Guestrin, 2016), SHapley Additive exPlanations (SHAP) (Lundberg & Lee, 2017), and Recursive Feature Elimination (RFE).

While the signals used for training are synthetic, this approach follows a “bottom–up” data-driven logic where the importance of each acquisition feature is learned from the statistical observation of one million noisy signal instances. By training on such an extensive dataset, the framework captures the intricate non-linear relationships and degeneracies inherent to the NEXI model under realistic noise conditions, rather than relying solely on local analytical derivations.

However, the complexity and opacity of many modern models, particularly gradient-boosted ensembles, can hinder interpretability, which is essential for clinical trust and scientific insight. XGBoost is especially well suited to this task due to its high performance on tabular data and robustness to noise, but while individual decision trees are interpretable, large ensembles are complex. We address this using fast post-hoc explanation methods (Sagi & Rokach, 2021), where SHAP is the key component. By leveraging cooperative game theory, SHAP assigns consistent importance values to each acquisition condition by accounting for the interaction between features. Each unique combination of b-value and diffusion time is considered a feature, which RFE then progressively prunes based on these SHAP rankings. Together, this XGBoost-SHAP-RFE pipeline, hereafter referred to as XAI, implements an explainable, scalable approach to acquisition design, with successful applications in medical imaging (Rufino et al., 2024; Zhu et al., 2025), neuroscience (Huang et al., 2024), and even non-biomedical domains such as environmental monitoring (Wang et al., 2025), cybersecurity (Ahmed et al., 2024), and user modeling (Chernyaeva & Hong, 2023). It is noteworthy that other approaches such as polynomial regression are also interpretable Coelho et al. (2024); however, XAI handles non-linear interactions and noise distributions without assuming a specific functional form for the error surface.

In this study, we extend this approach to diffusion MRI. We train XGBoost models on synthetic NEXI signals and use SHAP-based RFE to identify the smallest protocol subset that preserves estimation accuracy for *t_ex_*, *f*, *D_i_*, and *D_e_*. We benchmark this protocol against a theoretically optimal Weighted CRLB design and two expert-designed heuristic baselines (representing standard “Mid-Range” and extreme “Corner” sampling strategies). We validate it in vivo on Connectome 2.0, and assess its fidelity, reproducibility, and adaptability to higher-resolution imaging. This resolution, while not at the absolute limit of what is possible, is considered high for complex diffusion models and represents a significant step toward addressing partial volume effects in cortical gray matter. Our results show that simulation-based data-driven optimization not only reduces scan time to 14 minutes (vs. 27 minutes for the full protocol, and 30–40 minutes for previous NEXI protocols, Uhl et al. (2024b, 2025)), but also converges with the theoretical optimum while significantly outperforming heuristic designs in preserving biologically relevant contrast and parameter stability.

Ultimately, this work highlights the promise of interpretable machine learning for advancing diffusion model design and making advanced microstructural imaging more accessible in both research and clinical contexts.

## 2 Methods

### 2.1 Participants

Seven healthy adult volunteers (three males, mean age 29.9 ± 4.1 years) were scanned under informed consent in accordance with institutional ethical guidelines. Each participant underwent two identical scan sessions on the Connectome 2.0 MRI system, performed one after the other, to evaluate test–retest reproducibility of NEXI parameter estimation.

### 2.2 MRI acquisition

All imaging was performed on a MAGNETOM Connectom.X 3T scanner (Siemens Healthineers) equipped with a 500 mT/m gradient amplitude and 600 T/m/s slew rate (Ramos-Llordén et al., 2025), using a 72-channel head coil (Mahmutovic et al., 2025). Diffusion-weighted imaging (DWI) was acquired with a single-shot spin-echo EPI sequence and monopolar gradient pulses. Imaging parameters were voxel size = 2 mm isotropic, repetition time (TR)/echo time (TE) = 3300/66 ms, matrix size = 96 × 96, 72 axial slices, partial Fourier = 6/8, in-plane acceleration = 2, and simultaneous multislice (SMS) acceleration = 2.

The full protocol was a comprehensive acquisition scheme designed heuristically to ensure broad sampling of the acquisition parameter space. It consisted of 15 (*b*, Δ) combinations, spanning b-values from 1.0 to 12.5 ms/µm^2^ and diffusion gradient separations Δ from 12 to 45 ms, with a fixed gradient duration δ = 5 ms (where the effective diffusion time is defined as $t_{d}\;=\Delta -\delta /3$ ). The restriction to relatively short diffusion times (Δ≤45 ms) is driven by the trade-off between exchange sensitivity and signal-to-noise ratio (SNR). Extending Δ necessitates a longer TE, which drastically reduces SNR due to *T*_2_ relaxation and compromises the stability of the fit. A maximum Δ of 45 ms was selected as the optimal compromise. Directional sampling ranged from 20 to 64 directions depending on b-value (20 directions at *b* = 1, 30 at b=2.5 , 32 at *b* = 5, 34 at b=7.35−7.50 , 44 at b=10.0 , and 64 at b=12.5 ms/µm^2^). The diffusion time needs to be fixed for each diffusion MRI protocol. Data acquisition was thus split across 5 different sequences, 1 for each Δ. Each scan also included non-diffusion-weighted images (*b* = 0), totaling 13 volumes. The number of diffusion directions per shell was scaled heuristically with the b-value (approximately following a square-root dependence, $N_{dir}\;\propto \sqrt{b}$) to partially compensate for the expected drop in SNR at higher diffusion weightings, thereby harmonizing the SNR across shells. Non-diffusion-weighted images (*b* = 0) were acquired at each distinct Δ value to serve as specific reference signals, allowing for block-wise normalization to mitigate potential signal drifts or inter-sequence motion. Total scan time was approximately 27 minutes (see Table 1).

**Table 1.** Details of the full acquisition protocol.

b-Value $\left(\text{ms}/\mu m^{2}\right)$	Directions ($N_{\text{dir}}$ )	Diffusion time Δ (ms)
1.0	20	12, 27, 45
2.5	30	12, 27, 45
5.0	32	15, 27, 45
7.35 / 7.5	34	20, 27, 45
10.0	44	27, 45
12.5	64	45

Note: b = 0 images were acquired for each Δ.

Summary of b-values, number of diffusion directions per shell ($N_{\text{dir}}$), and associated diffusion times (Δ). The gradient duration is fixed at δ = 5 ms for all acquisitions.

This specific experimental design formulates the optimization problem as a discrete subset selection task (feature elimination) from a master protocol, rather than a continuous optimization of (*b*, Δ) coordinates. While continuous optimization theoretically allows for finer tuning, implementing arbitrary unique Δ values on clinical scanners typically introduces significant logistical overhead, often requiring separate sequence executions for each timing. By optimizing a subset of a feasible master protocol, we ensure immediate translational applicability.

The reduced protocol retained 8 features selected via a SHAP-based feature elimination strategy (see below), with 250 directions in total and identical *b* = 0 sampling. Acquisition time for the reduced protocol was 14 minutes. The 8 selected features spanned a broad range of b-values and diffusion times: (b,Δ) pairs of (1.0, 12), (2.5, 12), (5.0, 15), (7.3, 20), (1.0, 27), (1.0, 45), (2.5, 45), and (12.5, 45) ms/µm^2^ and ms, respectively. Both protocols were pre-processed and processed independently to allow for a matched comparison.

To enable cortical segmentation, a high-resolution T1-weighted anatomical scan (MPRAGE) was collected during each session with 0.9 mm isotropic resolution (TR/TE = 2500/2.9 ms, TI = 1100 ms, flip angle = 8°).

To preliminarily assess the protocol’s robustness to partial volume effects, an additional higher-resolution acquisition was performed using the reduced eight-feature protocol at 1.6 mm isotropic resolution for one last participant. To compensate for the lower SNR at this resolution, the total number of diffusion directions was increased to 1,000 across all shells (including 3 × *b* = 0, 80 directions each at *b* = 1 and b=2.5 , 128 at *b* = 5, 136 at b=7.35 , and 256 at b=12.5 for each Δ), the repetition time needed to be extended to TR = 4100 ms and the matrix size 138 x 138 with 82 axial slices. As a result, the scan duration increased 4-fold, and the mean SNR decreased only moderately, from 32 (Confidence Interval (CI): [13, 60]) at 2 mm to 27 (CI: [11, 56]) at 1.6 mm. This 69-minute scan enabled exploratory assessment of spatial fidelity and model robustness at finer anatomical scales.

### 2.3 Preprocessing

For each protocol, all diffusion-weighted volumes were jointly preprocessed using a standardized pipeline. This process began with complex-domain Marchenko-Pastur Principal Component Analysis (MP-PCA) denoising (Veraart et al., 2016). To correct for slowly varying phase variations, a two-pass procedure was employed: an initial MP-PCA pass was run on the complex data, the low-pass filtered phase from this output was used to correct the original data’s phase, and a final MP-PCA pass was applied to the phase-corrected data. The real part of this final result was used for subsequent steps. The pipeline then continued with Gibbs ringing correction (Kellner et al., 2016), susceptibility-induced distortion, and eddy current correction via FSL’s topup and eddy tools (Andersson & Sotiropoulos, 2016), and finally, gradient nonlinearity correction using scanner-specific maps (Huang et al., 2021).

*T*_1_-weighted images were segmented using FastSurfer (Henschel et al., 2020), and gray matter regions of interest (ROIs) from the Desikan–Killiany–Tourville (DKT) atlas (Klein & Tourville, 2012) were projected from *T*_1_ to diffusion space via affine registration with ANTs (Avants et al., 2009).

### 2.4 Model fitting

For all protocols, powder-averaged diffusion signals were calculated and NEXI parameters were estimated using the same nonlinear least-squares optimization framework (Jelescu et al., 2022) to ensure a fair comparison of the information content intrinsic to each acquisition scheme. Estimated parameters included intra-neurite diffusivity (*D_i_*), extra-neurite diffusivity (*D_e_*), intra-neurite signal fraction (*f*), and inter-compartment exchange time (*t_ex_*). Fitting constraints were applied to maintain biological plausibility and fitting stability: parameters were bounded within [1, 150] ms for *t_ex_*, [0.1,3.5] µm^2^/ms for both *D_i_* and *D_e_*, and [0.1,0.9] for *f*. Notably, the upper bound for *D_i_* was chosen slightly above free water diffusivity at body temperature, to leave margin for over-estimation due to CSF partial volume effects (which come with flow/pulsation artifacts) or noise. These constraints were consistent with previous implementations on 3T Prisma and Connectome 1.0 MRI systems (Uhl et al., 2024c, 2025). Cortical surfaces and parameter maps were visualized using Connectome Workbench (Marcus et al., 2011). To ensure statistical robustness and avoid kurtosis bias introduced by averaging signals with different underlying microstructural orientations, the model was fitted strictly at the voxel level. Any region-of-interest (ROI) mean signals or aggregated parameter maps presented in this study represent the subsequent averaging of these individual voxel-wise fits, rather than a single fit to a pre-averaged signal.

### 2.5 Synthetic simulations and protocol reduction

A synthetic dataset of 10^6^ diffusion signals was generated with the NEXI model. Ground-truth parameter vectors were sampled uniformly within empirical bounds obtained from the full 15-feature protocol (*t_ex_*:[1,70]ms, *f*:[0.15,0.80], *D_i_*:[1.7,3.5]ms/µm^2^, *D_e_*:[0.5,1.5]ms/µm^2^). These empirically derived ranges ensured that the simulations covered only physiologically plausible values while avoiding extreme outliers observed in low SNR voxels. Gaussian noise was then added so that the resulting SNR matched the in vivo distribution (mean:32, CI:[13, 60]). The four NEXI target parameters were z-scored before training, meaning we subtracted the mean and divided by the standard deviation for each parameter. This step ensures all parameters are on a common scale, which is essential for providing fair, unitless feature attributions. To demonstrate the scalability of our approach beyond the discrete 15-feature master protocol, we also performed an extended optimization on a high-density, physically constrained (*b*, Δ) grid, the details of which are provided in the Supplementary Materials (Supplementary Fig. S8).

An XGBoost regression model (Chen & Guestrin, 2016) was trained to predict each parameter from the full 15-feature input. Feature relevance was quantified using SHAP (Lundberg & Lee, 2017), and RFE was applied to iteratively remove the least informative features.

The final eight-feature protocol was selected based on (i) the inflection point in normalized root mean square error (RMSE) across parameters and (ii) protocol compatibility with time-dependent diffusion kurtosis analysis (Aggarwal et al., 2020; Fieremans et al., 2010; Novikov et al., 2011; Solomon et al., 2023) for secondary validation of exchange effects.

#### 2.5.1 Implementation details

The XGBoost models were trained with 128 trees, a maximum depth of 8, a learning rate of 0.3, a subsampling ratio of 0.6, and an ℓ_2_-regularization coefficient (lambda) of 1.0. These hyperparameters were determined through a grid search optimization on a validation set to minimize the mean squared error while preventing overfitting. Training was stopped early if the validation loss failed to improve for 25 consecutive boosting rounds. While the initial one-million synthetic signals were generated uniformly to ensure robust training (split into 80% training and 10% validation examples), the remaining 10% hold-out test examples were resampled to match the realistic in vivo distribution derived from the full protocol results. This ensures that the final performance benchmarking (e.g., Fig. 3) reflects biologically plausible conditions rather than a uniform distribution that would over-represent unlikely physiological regimes. Feature attributions were computed with the tree-SHAP algorithm implemented in the shap Python library https://github.com/shap/shap. Recursive feature elimination proceeded by removing the least important feature at each iteration until only two features remained. To ensure reproducibility and provide a benchmark for future users, the pipeline was executed on a laptop equipped with an Intel Core i7-13700H (16 cores), 30 GB of RAM, and an NVIDIA GeForce RTX 4070 Laptop GPU. Using GPU acceleration for XGBoost, the initial training on 1,000,000 samples was completed in 5.05 seconds, and the entire recursive optimization process (XGBoost-SHAP-RFE) was finalized in 2 minutes and 46 seconds. We inspected the resulting normalized RMSE curve and retained the eight-feature subset located near its inflection point. All scripts, trained models, and the synthetic signal generator are available in the repository cited in the Data and Code Availability section.

### 2.6 Comparison of protocol performance

To benchmark the performance and robustness of the XAI-optimized protocol against both theoretical limits and common expert practices, we evaluated it against the full 15-feature acquisition and 3 alternative 8-feature subprotocols:

- **Theory-driven Weighted-CRLB:** We implemented a rigorous optimization based on the CRLB. For each simulated ground-truth voxel, the Jacobian matrix was computed and scaled by the corresponding noise standard deviation per shell. To account for the uneven number of directions per shell, the noise variance term in the CRLB calculation was appropriately scaled for each shell as $\sigma_{\text{shell}}^{2}\;=\sigma_{\text{voxel}}^{2}\;/N_{\text{dir}}$ , ensuring that shells with more directions were correctly weighted as having higher precision. The inverse of the resulting FIM provided the local CRLB covariance matrix. To avoid bias from the different orders of magnitude and numerical ranges of the NEXI parameters (e.g., $t_{ex}\;\in \left[1,150\right]$ ms vs. f∈[0.1,0.9]), we used a weighted A-optimality criterion. More precisely, the diagonal elements of the CRLB matrix, representing parameter variances, were normalized by the square of their respective ground-truth parameters, converting them into squared coefficients of variation. We then minimized the trace of this weighted covariance matrix, averaged across the entire physiological prior distribution. This protocol represents the theoretical gold standard under Gaussian noise assumptions.
- **Heuristic “Mid-Range”:** This protocol mimics a standard multi-shell acquisition compatible with models such as SANDI but with an additional Δ. It densely samples b-values at a single intermediate diffusion time (Δ = 27 ms), supplemented by limited corner points (b=1,2,12.5 ms/µm^2^) at Δ = 45 ms. This design prioritizes b-value coverage but lacks temporal diversity.
- **Heuristic “Corner”:** Responding to the design principle of maximizing the lever arm, this protocol samples only the extremes of the acquisition space: the lowest and highest b-values at the shortest and longest Δ. While theoretically maximizing sensitivity to signal decay, this approach is potentially vulnerable to noise at high b-values.

The comparison across all protocols was multifaceted. In simulations, we assessed the absolute estimation error. For the in vivo data, comparison metrics included the root mean squared deviation (RMSD) of region-of-interest (ROI) medians between protocols, the similarity of parameter distributions across the cortex, and the visual agreement of the resulting cortical surface maps.

Test–retest repeatability was assessed by computing Bland–Altman plots of ROI medians across repeated sessions for each protocol. Limits of agreement and estimation bias were calculated separately for all four NEXI parameters.

## 3 Results

### 3.1 Cortical mapping with the full protocol

Figure 1 shows group-averaged cortical maps of NEXI parameters estimated from the full 15-feature protocol. As previously reported (Chan et al., 2025; Uhl et al., 2024c, 2025), elevated values of *t_ex_* and *f* are observed in the sensorimotor cortex, suggesting increased neurite packing and myelination. Interestingly, high *t_ex_* values are not observed in visual or temporal regions, unlike prior studies on Connectome 1.0 and Prisma. Our finding for the visual cortex also contrasts with a previous report on the Connectome 2.0 system (Chan et al., 2025). Instead, high *f* values are estimated in the temporal lobe. The intra-neurite diffusivity *D_i_* remains largely homogeneous across the brain, often hitting the enforced upper bound (3.5 µm^2^/ms), while *D_e_* is slower in high-*f* regions, consistent with reduced extracellular space.

**Fig. 1.**
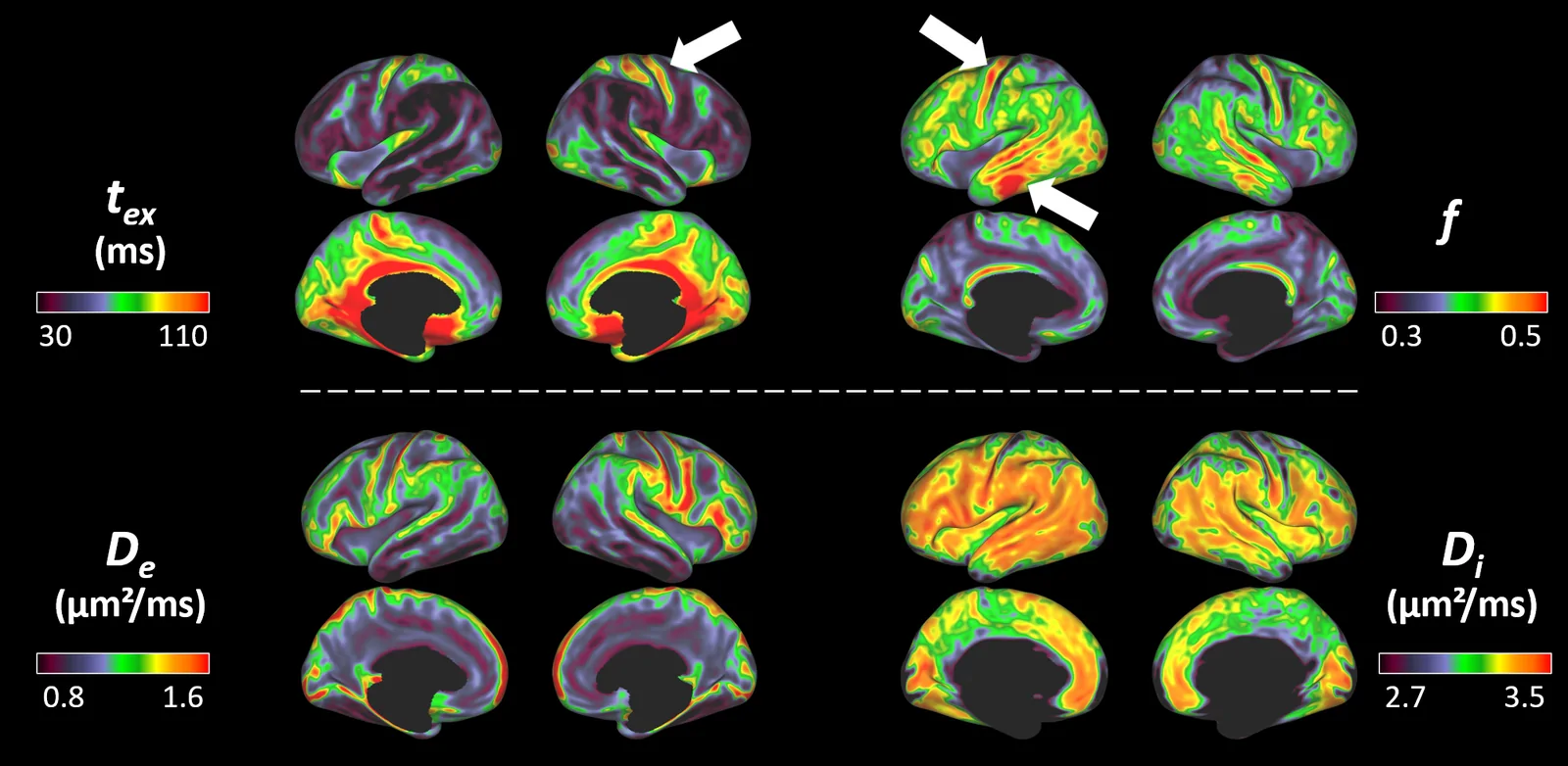
Cortical surface maps of NEXI parameters from the full protocol. Group-averaged maps of *t_ex_*, *f*, *D_i_*, and *D_e_* across all subjects, displayed on lateral and medial views of both cortical hemispheres. As expected, *t_ex_* and *f* are elevated in the motor cortex, consistent with prior associations with increased myelination and neurite density. However, unlike earlier reports on Connectome 1.0 and Prisma systems, no clear elevation of *t_ex_* is observed in the visual cortex or temporal lobe. Interestingly, *f* is elevated instead in the temporal lobe. In contrast, *D_i_* remains relatively uniform across the cortex (3 µm^2^/ms), while *D_e_* appears reduced in areas with high *f*, potentially reflecting more restricted extracellular environments. These full protocol maps are used as the spatial reference for evaluating the fidelity of reduced protocols. Arrows highlight the elevation of *t_ex_* and *f* in the motor cortex, as well as the elevation of *f* in the temporal lobe.

### 3.2 Data-driven protocol reduction using XAI

To identify a reduced acquisition protocol, we applied a machine-learning-based recursive feature elimination strategy using SHAP values computed from XGBoost regressors trained on synthetic NEXI signals. Figure 2 outlines this process. It revealed an inflection point at seven features in the root mean squared error (RMSE) curves, beyond which additional features yielded marginal improvements (Fig. 2). The primary motivation for retaining this specific additional acquisition (b = 1ms/µm^2^ at a short Δ) is that it allows for the estimation of diffusion kurtosis at a second diffusion time, thereby enabling time-dependent DKI analysis (Fieremans et al., 2010; Novikov et al., 2011) and a secondary measurement of *t_ex_* beyond the NEXI model itself. To rigorously assess the stability of this feature ranking, we repeated the entire XAI-RFE optimization process using 15 different random seeds. In every instance, the pipeline converged to the exact same eight-feature protocol. This confirms that the feature selection is highly robust to initialization variability and reflects the underlying physical geometry of the signal rather than stochastic noise in the training process. The retained features covered a broad range of b-values (1.0–12.5 ms/µm^2^) and diffusion times (Δ from 15–45 ms), ensuring sensitivity to both restricted diffusion and exchange effects.

**Fig. 2.**
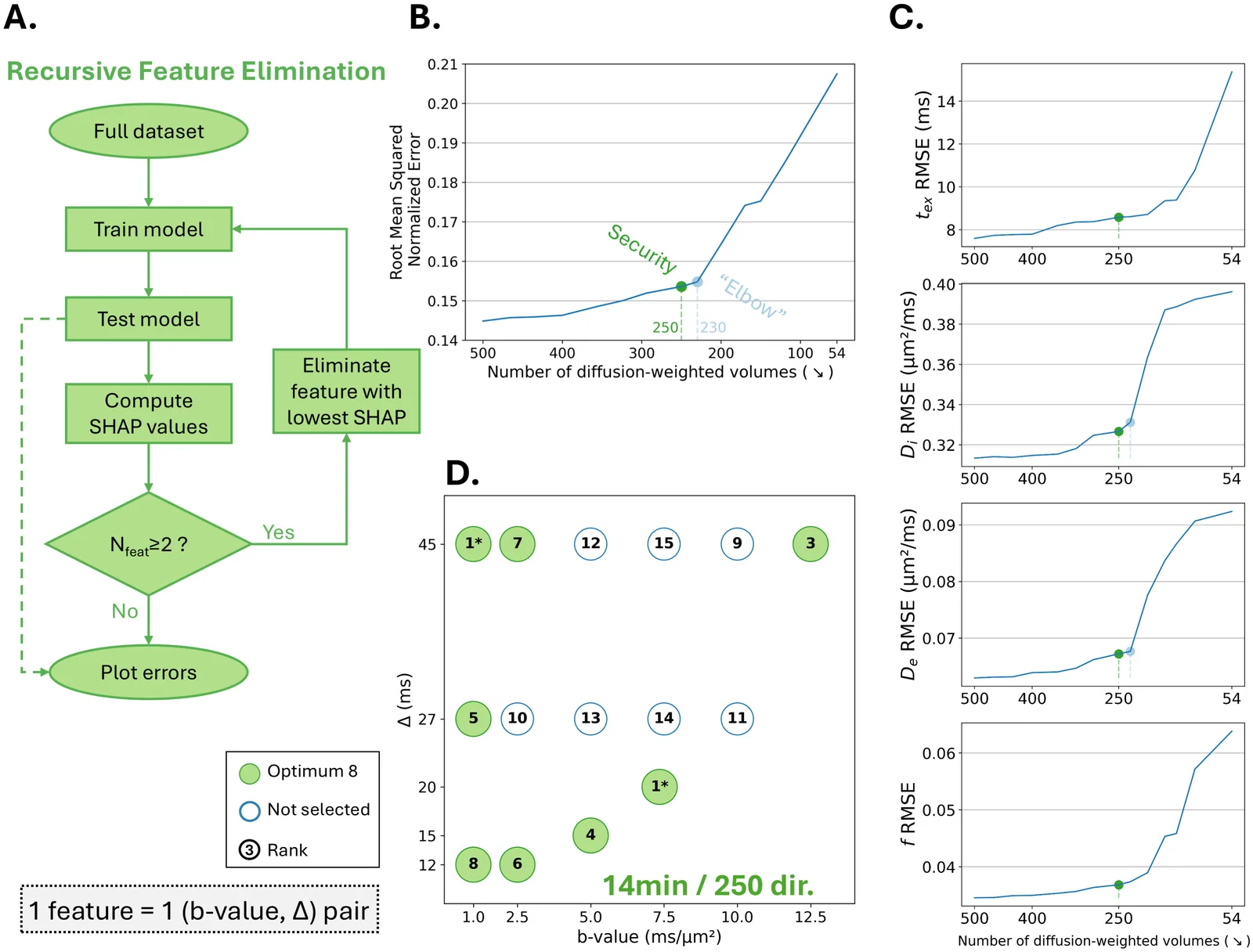
Recursive feature elimination using SHAP for NEXI protocol optimization. (A) Overview of the XAI-RFE pipeline: simulated NEXI signals were used to train XGBoost regressors, and SHAP values quantified each feature’s contribution to parameter estimation. Features (defined by unique *b* and Δ values) were iteratively removed based on minimal SHAP importance. Evolution of normalized RMSE across all (B) and each (C) parameters as features (and thus diffusion-weighted volumes) is removed. An inflection at 7–8 features (totalizing 230–250 diffusion-weighted volumes across the remaining features) indicates the minimal protocol length before accuracy sharply degrades. (D) Visualization of the final selected protocol (green), overlaid on the full 15-feature set. The retained features span both short and long diffusion times and a broad range of b-values.

### 3.3 Validation on synthetic signals and in vivo distributions

Validation on 100,000 synthetic noisy signals (SNR = 32 ± 26 at b = 0) (hold-out set, not used during any stage of the XGBoost training or RFE process) demonstrated that the XAI-reduced protocol preserves estimation accuracy across the full physiological range of NEXI parameters (Fig. 3). A consistent overestimation of *t_ex_* is observed for intermediate values, likely related to limited sensitivity at short Δ. *D_i_* tends to be underestimated at intermediate values. Despite this, the reduced protocol shows estimation profiles nearly identical to the full acquisition. Paradoxically, the reduced protocol can even marginally outperform the full protocol in specific error bins (e.g., for *f*). This slight improvement occurs because discarding certain high b-value shells that are heavily dominated by noise effectively reduces the fitting instability for these specific parameters. Crucially, parameter estimation on this hold-out set was conducted using a standard non-linear least squares (NLS) fitting procedure rather than the XGBoost model used for optimization. This ensures that the identified eight-feature protocol is intrinsically informative and that its performance is not dependent on a specific machine-learning estimator.

**Fig. 3.**
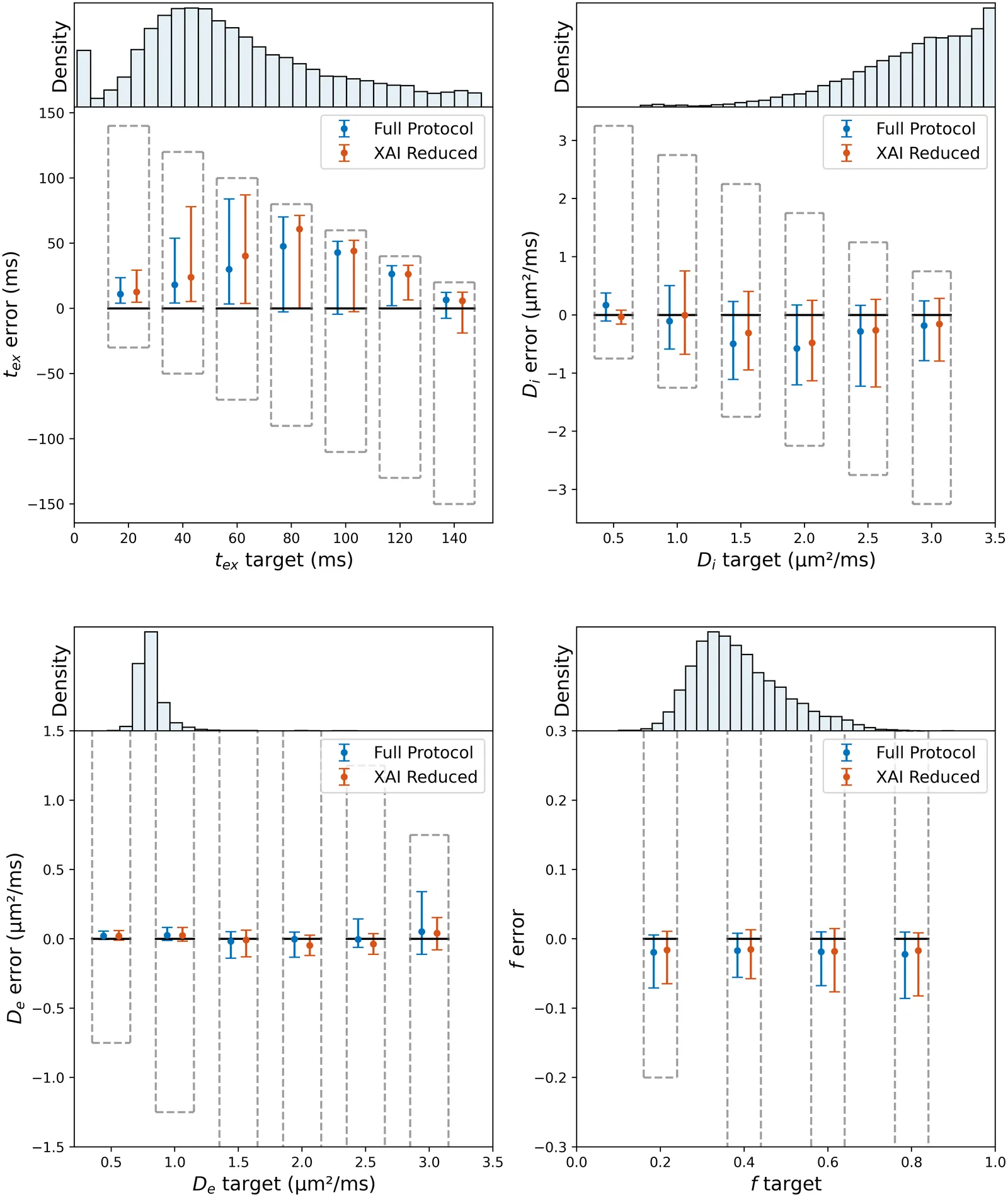
Estimation error across the parameter space: Full vs. XAI-optimized protocol. Binned error analysis from 100,000 synthetic NEXI signals with added Gaussian noise (SNR = 32 ± 26), comparing the full protocol (blue) and the reduced 8-feature XAI protocol (orange). Parameters were estimated using a classical non-linear least squares (NLS) solver for both the full (blue) and reduced (orange) protocols. For each NEXI parameter (*t_ex_*, *f*, *D_i_*, *D_e_*), absolute estimation errors are plotted as a function of the ground-truth parameter value. Note that y-axis limits are scaled specifically for each parameter to optimize the visibility of smaller error margins. Histograms above each panel indicate the distribution of target values in the test set. Error bars represent mean ± standard deviation within each bin; dashed rectangles denote model fitting bounds. Both protocols show similar error profiles across the full physiological range. A systematic positive bias in *t_ex_* is observed, reaching 20–40 ms for intermediate values, likely due to model limitations at short diffusion times or imperfect sensitivity to fast exchange. Estimates of *D_i_* tend to be underestimated at intermediate values. Errors for *f* and *D_e_* remain low and stable. These results confirm that the XAI-reduced protocol preserves parameter accuracy and robustness under realistic conditions.

Voxel-level distributions and regional medians in healthy volunteers confirm this consistency (Fig. 4). Although the reduced protocol yields a slightly heavier tail in the *t_ex_* distribution (as a trade-off to reducing the data points by 50%), modes and median values across DKT cortical ROIs show minimal differences between protocols, with *t_ex_* around 40 ms, f ≈0.36 , and $D_{e}\;\approx 0.9$ µm^2^/ms. These statistics (mode and median) are a priority for group-level analyses. These results, therefore, support the robustness of the XAI-reduced protocol across typical inter-subject variability.

**Fig. 4.**
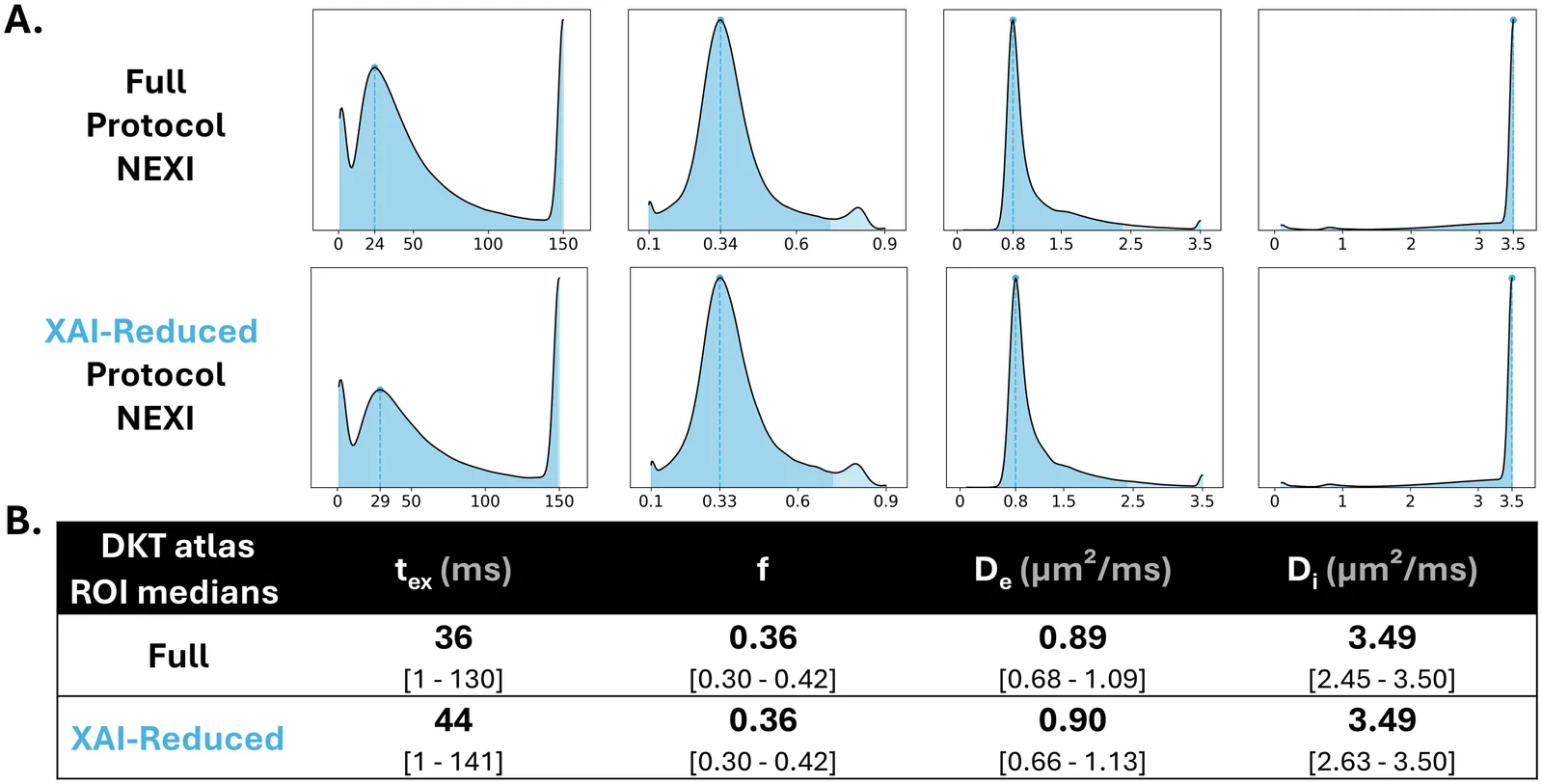
Distributions and summary of NEXI parameter estimates across DKT cortical regions. (A) Kernel density estimates of voxel-level values for each NEXI parameter (*t_ex_*, *f*, *D_e_*, *D_i_*), aggregated across all DKT ROIs and all subjects. The full protocol (top row) and XAI-reduced protocol (bottom row) yield very similar distributions. The vertical dashed lines indicate the mode of each distribution, reflecting the most frequent voxel value. Both protocols exhibit similar features: a strong ceiling effect for *D_i_* at the upper model bound (3.5 µm^2^/ms), right-skewed *t_ex_* distributions with local peaks around 25–30 ms, and unimodal distributions for *f* and *D_e_*. (B) Summary statistics computed from ROI-wise medians across the cortex. Median values and ranges are consistent between protocols, confirming the robustness of the XAI-reduced protocol.

### 3.4 Benchmark against theory-driven and heuristic protocols

To rigorously assess the optimality of the XAI-derived protocol, we benchmarked it against three alternative eight-feature subprotocols: a theoretically optimal protocol derived from the CRLB and two expert-designed heuristic protocols representing common acquisition strategies (Fig. 5).

**Fig. 5.**
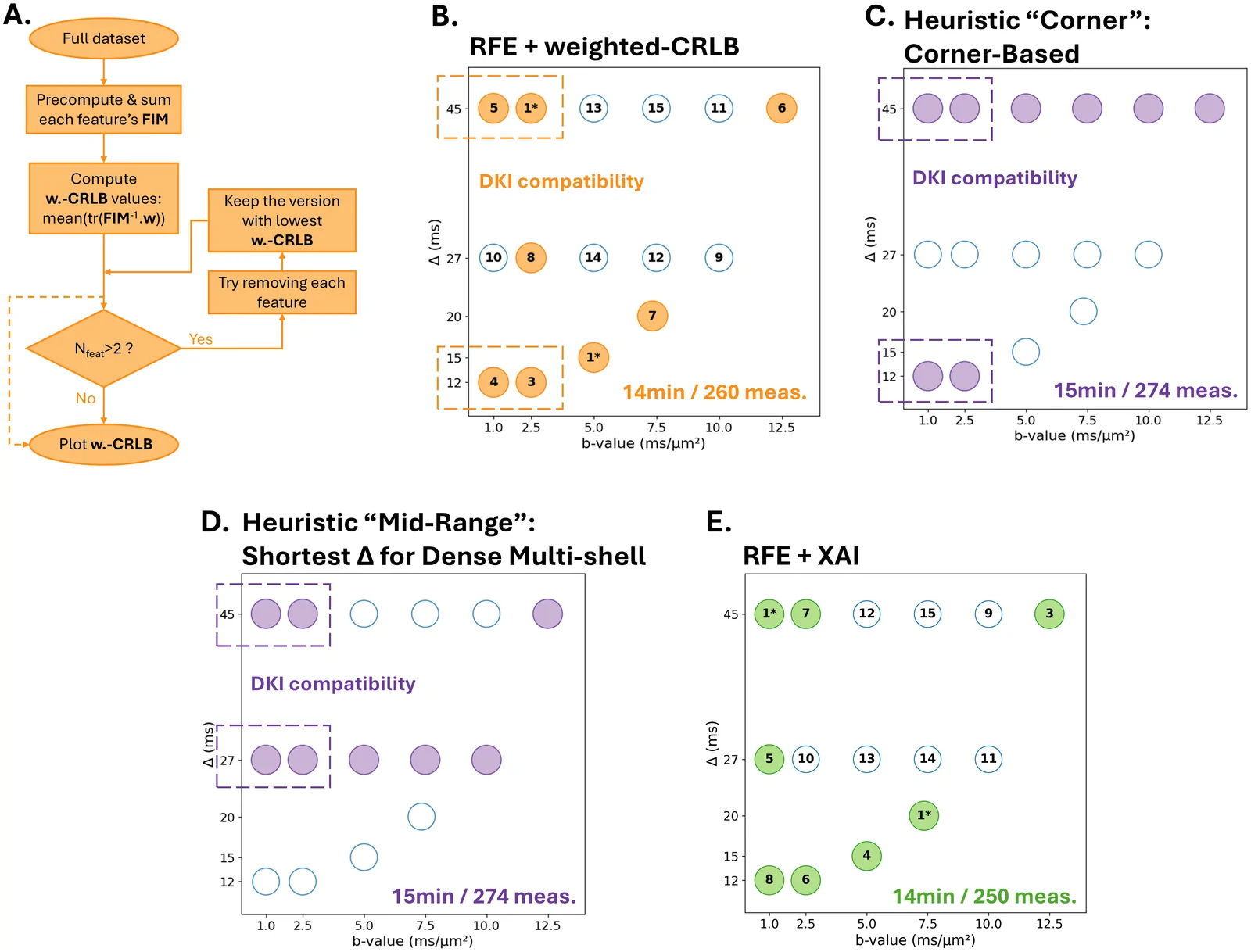
Design of theory-driven and heuristic comparison protocols. (A) Schematic of the Weighted CRLB-based Recursive Feature Elimination algorithm. The process iteratively removes features to minimize the mean relative variance (A-optimality) across a range of ground truths. (B) The resulting Weighted CRLB protocol is strikingly similar to the XAI-derived protocol, selecting features at the boundaries of the parameter space while maintaining temporal diversity. (C) The Heuristic “Corner” protocol samples only the extremes of the (*b*, Δ) space to maximize the geometric lever arm. (D) The Heuristic “Mid-Range” protocol densely samples a single intermediate diffusion time (Δ = 27 ms); this specific subset forms a standard multi-shell scheme compatible with fixed-diffusion-time models such as SANDI. The protocol adds only limited coverage at Δ = 45 ms, resulting in an overall lack of temporal spread critical for robust exchange mapping. (E) The XAI-optimized protocol (repeated for comparison) balances these constraints, achieving a configuration that mirrors the theoretical optimum (CRLB) while being derived purely from data-driven feature importance.

The theory-driven protocol was obtained using a weighted A-optimality criterion based on the CRLB. This method minimizes the sum of the relative variances (squared coefficients of variation) of the estimated parameters, averaged across a broad range of physiological ground truths. Remarkably, this purely theoretical optimization converged to a protocol nearly identical to the one identified by our data-driven XAI approach (Fig. 5B vs. E), providing strong independent validation of the XAI selection. Furthermore, to ensure these findings were not artifacts of the discrete 15-feature master protocol, both XAI and CRLB optimizations were extended to a high-density acquisition grid (Supplementary Fig. S8). Both methods consistently converged toward the same macroscopic strategy, confirming the robust nature of the derived protocol.

The two heuristic protocols highlight the pitfalls of intuitive design. The “Heuristic Corner” protocol (Fig. 5C) samples the extremes of the acquisition space to maximize the geometric lever arm. While theoretically sound for noiseless data, this approach proved highly unstable in practice. The “Heuristic Mid-Range” protocol (Fig. 5D) mimics a standard multi-shell acquisition with dense sampling at intermediate diffusion times (Δ = 27 ms), compatible with models such as SANDI, but lacks the temporal diversity required for robust exchange mapping.

Quantitative comparisons of in vivo estimations (Fig. 6) reveal that the XAI-optimized protocol consistently outperforms the heuristic alternatives. It yields the lowest root mean square deviation (RMSD) from the full protocol across all parameters. The “Heuristic Mid-Range” protocol exhibits a substantial bias in *t_ex_* (+33 ms) and a 2.4-fold higher RMSD compared with XAI, confirming that insufficient Δ sampling severely compromises exchange time estimation. Conversely, the “Heuristic Corner” protocol suffers from massive instability in *D_i_* (RMSD 0.79 vs. 0.11 μm^2^/ms for XAI) and a significant negative bias (-0.45 μm^2^/ms), demonstrating that extreme sampling is vulnerable to noise at high b-values. The weighted CRLB protocol performs comparably with XAI, though with slightly higher deviation in *f* and *D_i_*. These findings are supported by estimation errors from synthetic simulations and voxel-level distributions, which expose the noise sensitivity of the Corner strategy and the systematic biases of the Mid-Range strategy (Supplementary Figs. S1 and S2).

**Fig. 6.**
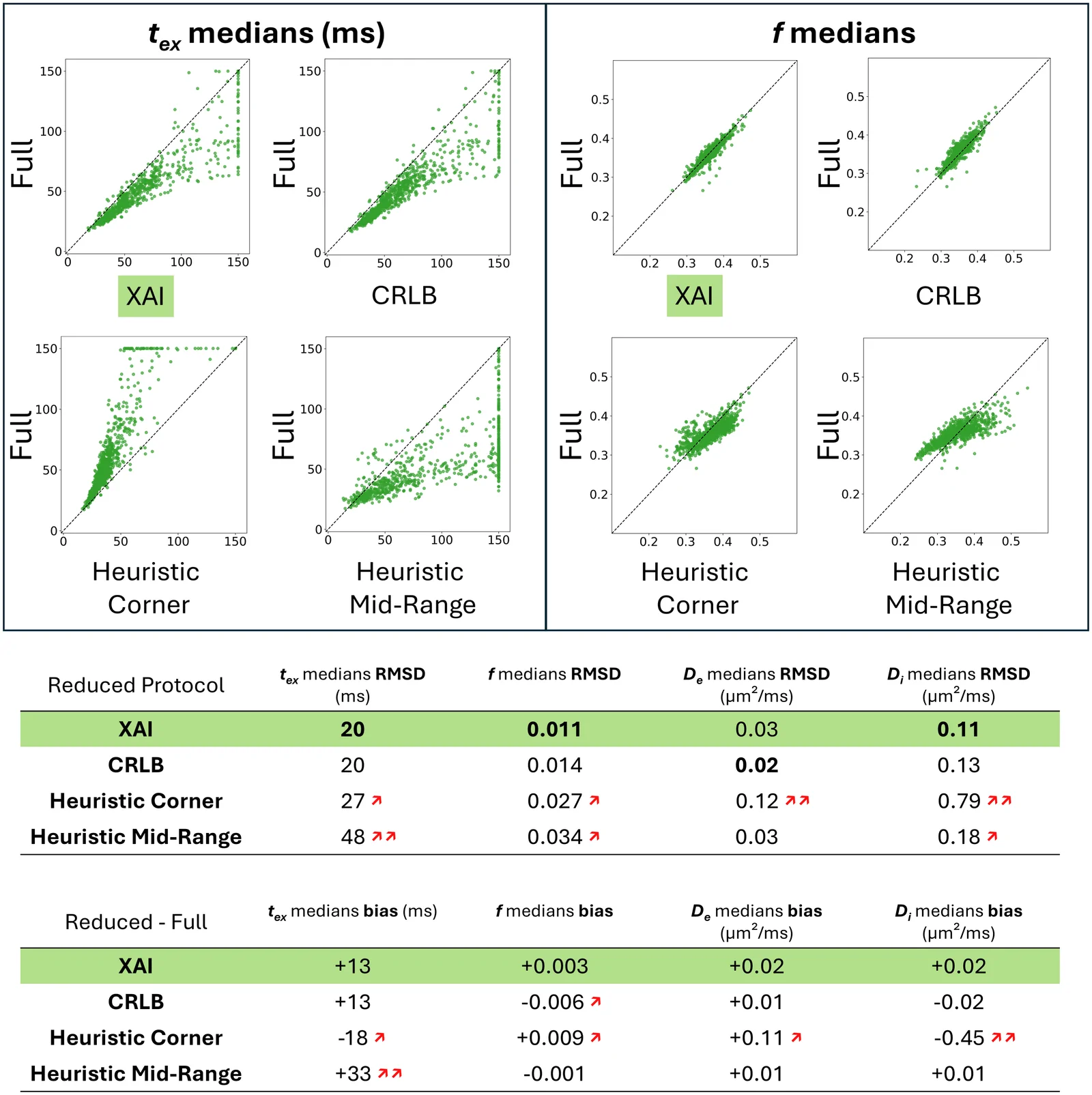
Quantitative benchmark: RMSD and estimation bias across protocols. Top: Scatter plots comparing DKT ROI medians from the full protocol (y-axis) against each reduced protocol (x-axis) for *t_ex_* and *f*, aggregated across all ROIs and subjects. The XAI and CRLB protocols show good agreement with the full acquisition (identity line). The “Heuristic Corner” protocol shows increased dispersion, while the “Heuristic Mid-Range” protocol exhibits a systematic bias for *t_ex_*. Bottom: Table summarizing the root mean square deviation (RMSD) and mean bias for all parameter ROI medians. Values in bold indicate the best performance. Red arrows highlight significant performance degradation compared with the XAI protocol. The XAI protocol achieves the lowest RMSD and bias overall. Notably, the “Heuristic Mid-Range” protocol shows a large bias in *t_ex_* (+33 ms) due to limited Δ diversity, while the “Heuristic Corner” protocol suffers from severe instability in *D_i_* (RMSD 0.79) and negative bias (-0.45), confirming its susceptibility to noise.

Surface parameter maps (Fig. 7) visually reinforce these quantitative findings. The XAI protocol faithfully reproduces the spatial contrasts of the full protocol, preserving the elevated *t_ex_* and *f* patterns in the sensorimotor and visual cortices. In contrast, the “Heuristic Mid-Range” protocol yields a flattened, over-smoothed *t_ex_* map that fails to capture regional variation, while the “Heuristic Corner” protocol produces a noisy and artifact-prone *D_i_* map, with extensive areas hitting the lower estimation bounds (dark regions). These results underscore that neither maximizing b-value range alone nor maximizing sampling density at a single Δ is sufficient; the XAI-identified sampling strategy provides the optimal compromise for robust microstructural mapping.

**Fig. 7.**
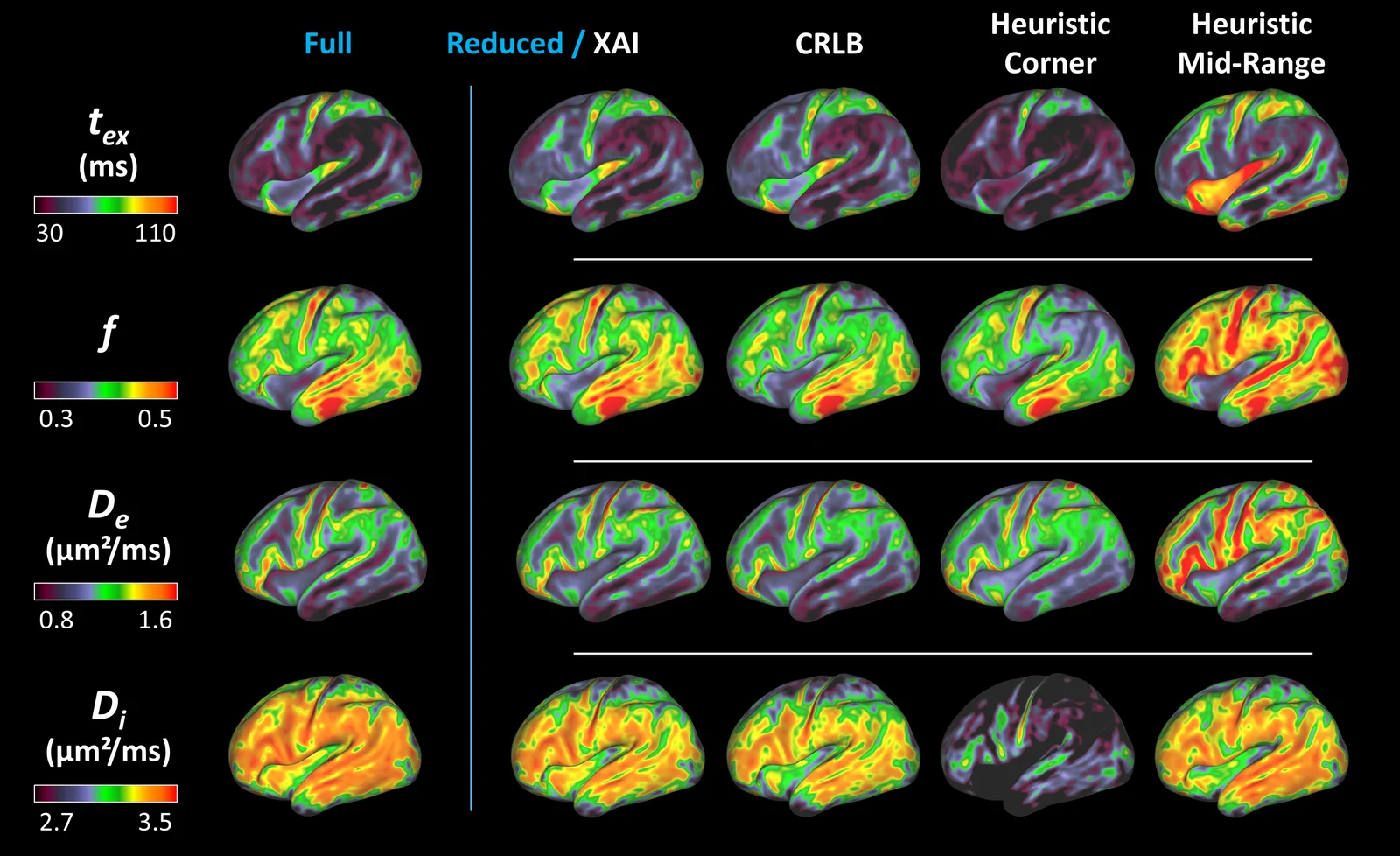
Cortical surface maps of NEXI parameters comparisons across protocols. Group-averaged maps for *t_ex_*, *f*, *D_e_*, and *D_i_* are displayed for the full protocol, the XAI-optimized reduced protocol, and the three benchmark protocols (CRLB, Heuristic Corner, Heuristic Mid-Range). The XAI protocol (second column) is visually indistinguishable from the full protocol (first column), preserving all key anatomical contrasts. The CRLB protocol also performs well but shows slightly more variance in *f*. The Heuristic Corner protocol fails catastrophically for *D_i_* (bottom row), producing a dark map indicative of underestimation due to noise sensitivity at high b-values. The Heuristic Mid-Range protocol yields a distorted *t_ex_* map (top row) with exaggerated values in the insular cortex and an increased contrast in the temporal lobe, confirming that insufficient diffusion time sampling biases exchange estimates.

### 3.5 Test–retest reliability and high-resolution scalability

Test–retest reproducibility was preserved with the reduced protocol. Bland–Altman analysis (Fig. 8) shows that the eight-feature protocol maintains agreement comparable with the full protocol across all parameters, with only marginal increases in the limits of agreement. The seemingly sparse distribution of data points in the *D_i_* Bland–Altman plots is a direct consequence of a strong ceiling effect: a large proportion of voxels consistently hit the upper model bound (3.5 µm^2^/ms) in both scan sessions, causing their coordinates to perfectly overlap on the limit line. In comparison, the alternative reduced protocols (see Supplementary Fig. S3) exhibited distinct trade-offs. While the Weighted CRLB protocol showed reproducibility profiles similar to the XAI protocol, the Heuristic Corner protocol demonstrated severe instability for *D_i_*. Conversely, the Heuristic Mid-Range protocol, though reproducible, yields the biased estimates detailed previously. This underscores that the XAI-optimized approach achieves the most robust balance between parameter stability and sensitivity across the full model space.

**Fig. 8.**
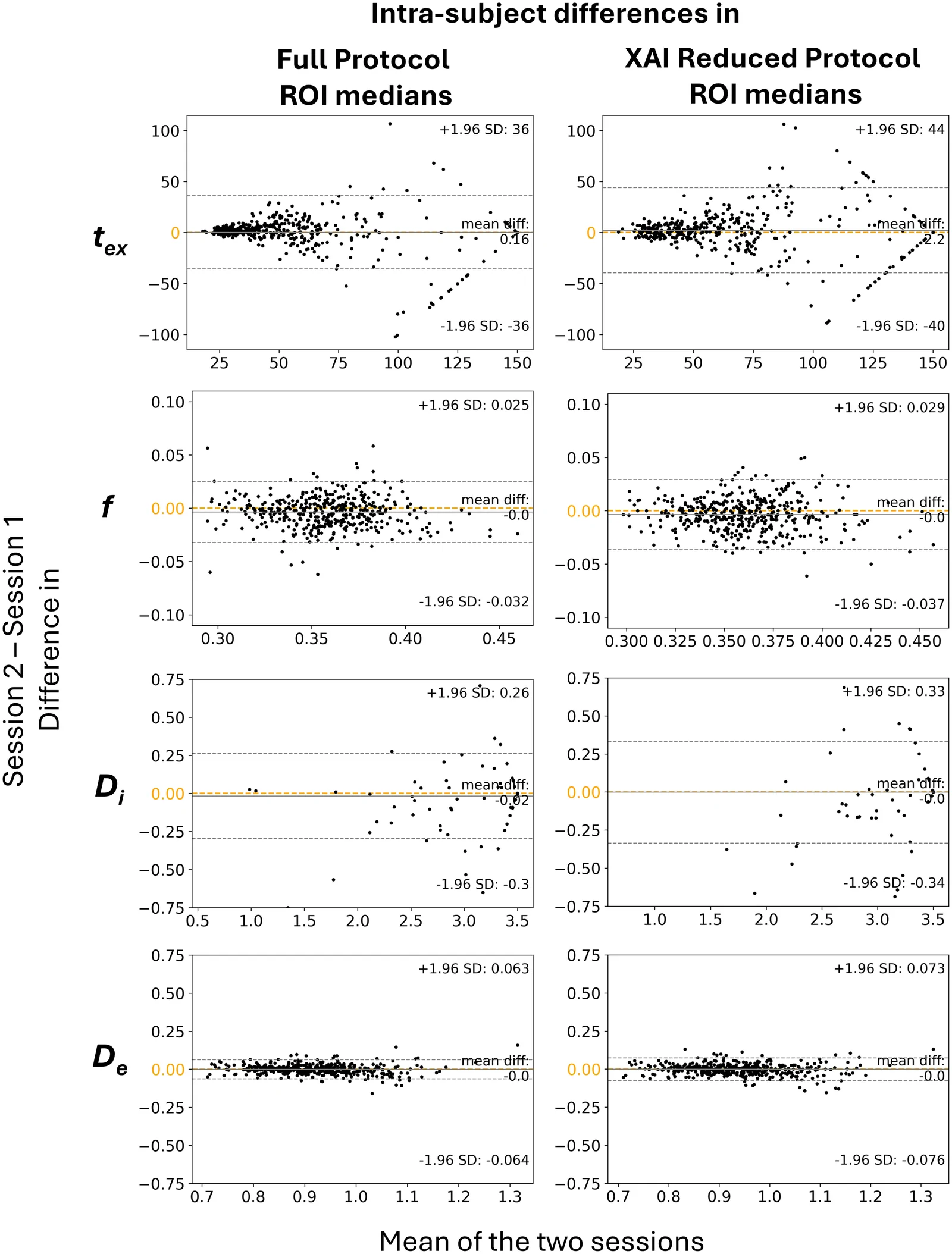
Test–retest reproducibility of NEXI estimates using the full protocol and the XAI-optimized subprotocol. Bland–Altman plots show intra-subject variability across scan–rescan sessions, using DKT ROI median values for each NEXI parameter (*t_ex_*, *f*, *D_i_*, *D_e_*). Left: Full protocol. Right: XAI-Reduced protocol. Each plot displays the difference between sessions against their mean, with orange lines indicating bias and dotted gray lines denoting the 95% limits of agreement. The XAI protocol maintains reproducibility comparable with the full protocol, with marginally wider limits for *t_ex_* and *D_i_* but near-identical performance for *f* and *D_e_*. This supports the use of the eight-feature reduced protocol in longitudinal studies where acquisition time is constrained.

To quantify stability, we computed the mean within-subject Coefficient of Variation (wsCV) for each parameter across all cortical ROIs (Table 2). The XAI protocol demonstrates excellent stability, nearly matching the full protocol’s performance. In stark contrast, the alternative reduced protocols exhibited distinct failure modes. While the Heuristic Mid-Range protocol yielded reproducible but biased estimates (as seen in Fig. 6), the Heuristic Corner protocol exhibited a polarized behavior. Paradoxically, it achieved the lowest wsCV for *t_ex_* (8.4%). While the XAI and CRLB protocols also favor the boundaries of the (*b*, Δ) parameter space, they critically maintain temporal diversity by retaining intermediate diffusion times (Δ=15,20,27 ms). In contrast, the Heuristic Corner protocol allocates its entire budget to the absolute extremes of Δ, heavily oversampling the longest diffusion time (Δ = 45 ms) while ignoring intermediate steps. This massive polarization maximizes the geometric lever arm for the exchange rate, artificially locking in the *t_ex_* estimate and reducing its variance. However, it lacks the intermediate temporal support needed to robustly capture the signal decay against noise at high b-values. Consequently, the wsCV for *D_i_* spiked to 5.7% in the Corner protocol, a nearly fivefold increase compared with the XAI-optimized protocol (1.3%) and the full acquisition (1.2%). This demonstrates that both data-driven and theory-based optimization effectively balance the trade-off between maximizing temporal leverage and preserving full model stability, a balance missed by extreme heuristic sampling.

**Table 2.** Quantification of test–retest reproducibility across acquisition protocols.

Protocol	$t_{\text{ex}}$ (%)	*D_i_* (%)	*D_e_* (%)	*f* (%)
Full protocol	10.9	1.2	1.8	2.2
XAI-reduced	12.8	1.3	2.1	**2.5**
CRLB-reduced	12.4	2.0	**1.9**	2.6
Heuristic Corner	**8.4**	5.7	2.0	2.6
Heuristic Mid-Range	12.2	**1.2**	**1.9**	2.9

Values represent the mean within-subject Coefficient of Variation (wsCV, in %) for each NEXI parameter, calculated across all subjects and cortical ROIs. The wsCV is defined as the standard deviation of paired scan–rescan measurements divided by their mean. We report the mean wsCV here specifically to expose the instability of the intra-neurite diffusivity (*D_i_*) in the *Heuristic Corner* protocol (5.7%) compared with the full protocol (1.2%). This instability, driven by noise sensitivity at high b-values, is masked when looking solely at median statistics (which are near 0% due to boundary constraints). Bold values indicate the lowest wsCV among the reduced protocols for each parameter.

Beyond protocol reduction, we explored the performance of the XAI-optimized protocol in higher-resolution settings. A 69-minute scan with 1.6 mm isotropic voxels was acquired in one subject, with an increased number of directions to retain the SNR. As shown in Supplementary Figures S5 and S6, spatial distributions of NEXI parameters remained qualitatively consistent with those at 2 mm, with enhanced delineation of the cortical ribbon and sharper gray–white matter boundaries. These findings indicate that the reduced protocol maintains pattern stability under increased spatial resolution, helping to mitigate partial volume effects in cortical gray matter.

We also evaluated the impact of scanner choice and denoising strategy. Cross-scanner comparisons (Supplementary Tables S1 and S2) revealed consistent *f* and *D_e_* estimates between systems, but longer *t_ex_* and elevated *D_i_* values on Connectome 2.0, likely due to increased sensitivity to fast intra-neurite dynamics enabled by higher gradient strength (Huang et al., 2021). However, *D_i_* frequently reaches the model’s upper bound, raising the possibility of unmodeled soma effects or fit instabilities.

Finally, we assessed the influence of denoising. Although complex MP-PCA denoising offers, in principle, a way to avoid Rician floor effects that confound signal interpretation at high b-values, magnitude-based processing with Rician correction currently yielded better model fits (Supplementary Fig. S4). This highlights the need for further refinement of complex-domain denoising methods tailored to high-gradient acquisitions.

## 4 Discussion

A fundamental barrier hinders the broader adoption of advanced diffusion models: the tension between their biophysical richness and the pragmatic need for time-efficient acquisitions. Optimizing the acquisition time would help promote wider adoption of NEXI in research and clinical applications. For this purpose, we leveraged a state-of-the-art research scanner, the Connectome 2.0, as it offers a unique capability to access a broader range of acquisition parameters enabled by its ultra-high gradient performance. This study successfully demonstrates the feasibility of robustly estimating Neurite Exchange Imaging (NEXI) parameters in human cortical gray matter using a substantially reduced diffusion MRI protocol consisting of only eight strategically selected (*b*, Δ) combinations. Leveraging a data-driven optimization framework based on explainable machine learning (XGBoost-SHAP-RFE or XAI), we achieved a twofold reduction in scan time on the Connectome 2.0 platform, from 27 to 14 minutes, while preserving key features of spatial contrast, parameter accuracy, and test–retest reproducibility. These findings provide a critical advancement toward the clinical and translational implementation of exchange-sensitive diffusion models, particularly in cortical regions where scan time constraints, noise susceptibility, and parameter degeneracy have traditionally hindered adoption.

### 4.1 Advantages of SHAP-guided empirical protocol reduction

The primary methodological contribution of this study lies in the use of SHAP-guided RFE to empirically optimize a heuristically designed diffusion protocol. Unlike conventional approaches based on the CRLB or the FIM (Flaherty et al., 2005; Jalnefjord et al., 2019), our method operates without relying on a purely theoretical framework. While the CRLB provides a rigorous lower bound on the local variance of an unbiased estimator, it struggles to account for physical boundary constraints (e.g., the strict upper bound on *D_i_*) and the non-linear estimation biases inherent to the fitting landscape. Even when extended to Rician noise (Karlsen et al., 1999), analytical optimization remains mathematically cumbersome and restricted to idealized contexts. By directly minimizing the global prediction error on synthetic data, our XAI framework naturally integrates the combined effects of realistic noise, signal correlations, and physical constraints. Furthermore, this model-agnostic approach bypasses the need for complex analytical Jacobians, making it easily adaptable to future models relying on numerical formulations or complex noise distributions.

A striking validation of this approach is the convergence we observed between the data-driven XAI selection and the theoretical weighted CRLB optimization. When the theoretical framework was properly normalized (using A-optimality on relative errors) to account for the disparate units of NEXI parameters, it selected a protocol nearly identical to the one identified by our XGBoost-SHAP-RFE pipeline. This convergence is significant for two reasons. First, it confirms that the XAI effectively learned the underlying information geometry of the biophysical model purely from simulated signal statistics. Second, it suggests that for the NEXI model in this acquisition regime, the presence of non-linear degeneracies does not fundamentally alter the optimal acquisition strategy. This mutual validation strengthens the credibility of the proposed eight-feature protocol, identifying it as a robust global optimum rather than an artifact of a specific selection algorithm. This convergence is further supported by an independent optimization using a Weighted D-optimality criterion based on the FIM rather than the CRLB, which yielded an identical protocol strategy (Supplementary Fig. S7).

This theoretical convergence raises a valid question regarding the added value of the XAI pipeline if it ultimately yields the same result as the CRLB. The primary advantage of the XAI approach lies in its model-agnostic flexibility. While calculating the CRLB is highly effective for analytical models such as standard NEXI under Gaussian noise, it becomes mathematically cumbersome or even entirely intractable for advanced numerical models lacking analytical Jacobians, or when dealing with complex, non-Gaussian noise distributions, such as Rician noise. In such scenarios, the XAI framework serves as a scalable alternative that relies purely on empirical prediction error rather than theoretical derivatives. In the context of this study, the XAI method acts as an independent benchmark, proving that machine-learning-based pruning can achieve analytical gold standards while remaining generalizable to future, more complex modeling frameworks.

The SHAP-RFE framework’s analysis revealed that the most informative features are located at the extremes of the (*b*, Δ) acquisition space. This is consistent with the need to sample a wide range of physical regimes to accurately constrain the biophysical model and prevent parameter degeneracy. This distinction proved crucial: standard heuristic or unweighted theoretical approaches often failed to capture the importance of including the longest diffusion time with the strongest b-value. This specific data point, which proved crucial for the accurate estimation of these parameters, was correctly identified as essential by our methodology.

It is worth noting that alternative optimization strategies, such as those proposed by Coelho et al. (2024) or Jespersen et al. (2025), could also be considered. These methods typically involve leaving a small, fixed number of acquisition points free to converge iteratively toward optimal positions in continuous parameter space (e.g., b-value). However, for NEXI, where we optimize across (*b*,Δ), this approach risks generating a large number of optimal points, each with a unique diffusion time. Since each unique Δ value often requires a separate sequence launch on the scanner, this diversity introduces a significant additional operational burden for the researcher or clinician running the protocol. Similarly, arbitrary b-values also limit the usability of the data for mainstream analyses such as DTI/DKI fits, for which a consistent choice of b-values improves cross-site harmonization substantially. By contrast, our SHAP-RFE framework selects a minimal subset from an existing, logistically constrained set of acquisition parameters, and, trained on synthetic NEXI signals spanning the full physiological range and realistic SNR regimes, yielded a reduced protocol that converged with the theoretical optimum derived from weighted CRLB while consistently outperforming expert heuristic strategies. This data-driven approach was chosen because it allows for a comprehensive and uniform sampling of the NEXI parameter space, which is critical for robust and unbiased training of the XGBoost regressor. The selected features span the full b-value range and preserve all Δ values, supporting estimation of time-dependent kurtosis. Notably, these combinations would be difficult to derive from intuition or analytic simplifications alone.

Our results confirm and extend the utility of SHAP-based RFE pipelines previously applied in other biomedical contexts (Rufino et al., 2024; Wang et al., 2025; Zhu et al., 2025). In particular, similar approaches using XGBoost-SHAP-RFE for feature pruning in physiological state classification (Huang et al., 2024), symptom triage (Rufino et al., 2024), and environmental sensor optimization (Wang et al., 2025) have demonstrated strong generalization under high-dimensional, noisy conditions or characteristics shared with diffusion MRI signal modeling.

In our context, the XGBoost-SHAP-RFE optimization led to an inflection point at seven features, beyond which accuracy gains plateaued (Fig. 2). However, deliberately retaining eight features ensured coverage of both short and long diffusion times, and allowed compatibility with secondary metrics such as time-dependent diffusion kurtosis (Henriques et al., 2019). Critically, the final protocol not only reproduced parameter estimates, but also preserved cortical contrast patterns observed in full acquisitions, including elevated *t_ex_* and *f* in sensorimotor cortex (Chan et al., 2025; Uhl et al., 2024c).

Finally, it is important to explicitly acknowledge the conceptual limitations of our chosen optimization strategy. By performing feature elimination on a predefined 15-feature master protocol, our framework identifies the optimal subset conditional on these available candidates, rather than solving a global joint optimization problem. In practice, MRI sequence design imposes coupled constraints: pushing for higher b-values or longer diffusion times requires a longer TE, which inherently reduces the base SNR of the entire acquisition due to *T*_2_ relaxation. By operating within a fixed master protocol where a single global TE is already established (e.g., TE = 66 ms), our approach treats the SNR and timing of each shell as fixed and independently selectable. It does not account for the potential SNR gains that could be achieved if eliminating the most demanding shells allowed for a sequence-wide TE reduction. Here we chose to operate under fixed TE to keep the problem centered on the diffusion-weighting parameter space. Indeed, SNR is also affected by other acquisition parameters such as spatial resolution, coils, and acceleration factors. The results of an optimization across a continuous parameter search (as explored in our high-density grid simulation in Supplementary Fig. S8) suggest that the optimal points are still on the edges of the (*b*, Δ) space accessible given a certain gradient hardware. Thus, restricting the search to a discrete, standard master protocol does not result in a very local optimum, while it ensures that the retained shells remain practical for implementing the protocol at the scanner and perfectly compatible with widespread multi-shell analytical techniques such as DTI and DKI.

### 4.2 Reproducibility and generalization of the reduced protocol

Beyond estimation accuracy, test–retest reproducibility is essential for longitudinal studies, particularly when subtle microstructural changes are of interest. We found that the XAI-reduced protocol maintained intra-subject consistency across repeated scans, with only marginal increases in limits of agreement for *t_ex_* and *D_i_* compared with the full protocol (Fig. 8). This aligns with prior findings that exchange-related metrics display moderate SNR sensitivity (Jelescu et al., 2022; Uhl et al., 2024c), but confirm that performance remains within ranges reported in prior studies on clinical and research scanners (Chan et al., 2025; Uhl et al., 2024c, 2025).

Importantly, the reduced protocol also demonstrated its generalizability to higher-resolution settings in an exploratory 1.6 mm isotropic acquisition performed on one subject. Acquiring data at this finer spatial scale presents a significant challenge due to the inherent reduction in SNR per voxel. To counteract this, we implemented a compensation strategy by substantially increasing the total number of diffusion directions to 1,000. In addition, the increased number of slices resulted in extending the repetition time (TR = 4100 ms) and thus further the scan time. While this necessarily resulted in a fourfold increase in scan duration to 69 minutes, this was a direct consequence of the physical trade-off required to maintain image quality: the voxel volume decreases by a factor of 2, necessitating a fourfold increase in the number of diffusion directions to compensate for the lost signal-to-noise ratio. This remains the optimal NEXI protocol for this high spatial resolution. Indeed, the equivalent full protocol would have lasted 138 minutes. The resulting parameter maps exhibited consistent topography and sharper gray–white matter boundaries compared with the 2 mm scans, suggesting that the XAI-optimized scheme remains robust enough to be extended to finer spatial scales and holds promise for future applications such as laminar-specific analyses at yet higher resolutions. These findings align with recent work advocating for resolution-adaptable diffusion protocols in cortex-focused imaging (Hertanu et al., 2023; Palombo et al., 2020).

Together, these results validate the use of explainable machine learning not merely as a black-box tool, but as a transparent and interpretable strategy for optimizing biophysically informed acquisition design. The flexibility of our framework allows retraining under alternative models (e.g., SMEX (Olesen et al., 2022), GEM (Uhl et al., 2024a)), scanner configurations, or populations of interest, thereby supporting future clinical translation and cross-site harmonization efforts.

### 4.3 Spatial specificity and reproducibility across the cortical ribbon

The spatial distributions of NEXI parameters obtained from the XAI-optimized protocol closely match those observed with the full acquisition, demonstrating that a data-driven subprotocol can preserve biologically relevant contrasts across the cortex. Elevated *t_ex_* and *f* in sensorimotor cortex, previously linked to dense myelination and high neurite packing (Chan et al., 2025; Uhl et al., 2024c, 2025), were clearly recapitulated. Interestingly, our data partially diverged from earlier studies by showing moderate *t_ex_* in visual cortex. This discrepancy may reflect increased sensitivity to fast exchange enabled by the short diffusion times of Connectome 2.0 (Δ=15−45 ms, δ = 5 ms) (Chan et al., 2025; Huang et al., 2021). We also observed unexpectedly higher *f* values in temporal regions, despite prior reports associating the temporal lobe with lower cellular and axonal density (Ribeiro et al., 2013), and previous NEXI maps consistently showing the opposite trend in this region (Chan et al., 2025; Uhl et al., 2024c, 2025). This may be explained by the lower SNR in these regions, which qualitatively affects the robustness of the parameter estimation.

In terms of estimation accuracy, the XAI-optimized protocol demonstrated a substantial improvement over the alternative schemes. RMSD calculations showed that the XAI protocol yielded a 2.4-fold lower RMSD in *t_ex_* than the Heuristic Mid-Range protocol, and avoided the severe instability in *D_i_* observed with the Heuristic Corner protocol (RMSD 0.11 vs. 0.79 µm^2^/ms). The XAI protocol consistently showed low RMSD and bias across all parameters, comparable with the theoretical CRLB optimum (Fig. 6). Importantly, test–retest analyses (Fig. 8) confirm that this compact protocol retains measurement reliability for longitudinal studies and sensitivity to between-subject differences. While *t_ex_* and *D_i_* showed marginally broader limits of agreement, their reproducibility remained within ranges reported in previous Connectome and Prisma studies (Uhl et al., 2024c, 2025). The stability of *f* and *D_e_* was particularly robust, supporting the feasibility of deploying this protocol in clinical cohorts where subject motion or compliance imposes strict scan-time constraints.

### 4.4 Limits of model fidelity and parameter identifiability

Our analysis also underscores persistent challenges in NEXI parameter estimation. Specifically, *D_i_* often hits its upper model bound (3.5 µm^2^/ms), a behavior also noted in prior work on white matter models such as NODDI (Howard et al., 2022; Jelescu et al., 2016) and in recent cortical applications of NEXI (Uhl et al., 2024c), suggesting that this lack of sensitivity to high intra-neurite diffusivity is a general feature of multi-compartment diffusion models. Importantly, while our synthetic simulations (Fig. 3) indicated a tendency for the estimator to underestimate *D_i_* at intermediate values, the systematic overestimation observed in vivo is likely driven by biological confounds not captured in the simulations, such as partial volume effects with cerebrospinal fluid, structural disorder, or the presence of unmodeled soma compartments. Similar concerns apply to *t_ex_* estimation, where we observe systematic overestimation at intermediate values (Fig. 3), likely arising from the interplay between limited short-Δ data and degeneracy in the fitting landscape. Despite these identifiability challenges at relatively short diffusion times (Δ≤45 ms), retaining the exchange component is essential to preserve the model’s primary biophysical value: probing membrane permeability. Our results indicate that the XAI-optimized protocol successfully maximizes the available information to constrain *t_ex_* within these hardware limitations.

Such biases raise important considerations for model selection and parameter interpretation. As shown in both simulation and experimental studies, models that neglect Rician noise can overestimate *t_ex_* (Chan et al., 2025; Uhl et al., 2024c), while neglecting unmodeled factors such as cell somas or structural disorder can introduce biases in the estimation of *D_i_*, *f*, and even *t_ex_* (Lee et al., 2020; Novikov et al., 2023). These observations echo similar issues reported in white matter modeling (Dhital et al., 2019; Jelescu et al., 2016) and point toward the need for more comprehensive signal models capable of handling gray matter complexity. The pronounced peaks at the parameter boundaries observed in the voxel-wise distributions (Fig. 4), specifically at the 3.5 µm^2^/ms limit for *D_i_* and the bounds for *t_ex_*, reflect these inherent modeling degeneracies. The inclusion of the highest b-value shell (12.5 ms/µm^2^) in both the XAI and CRLB optimal protocols perfectly exemplifies this tension: while it provides critical leverage for constraining *t_ex_*, it simultaneously renders *D_i_* more vulnerable to Rician noise floors, pushing it toward the upper bound. The XAI optimization navigates this necessary trade-off by minimizing the impact of these boundary effects on the overall normalized error, ensuring that key parameters such as *f* and *D_e_* remain robustly estimated.

Similarly, the finite duration of gradient pulses is known to inflate *D_i_* estimates, an issue addressed by numerical formulations such as SMEX (Olesen et al., 2022; Uhl et al., 2025). While our preliminary evaluations with SMEX on this dataset highly conserved spatial trends and mitigated the *D_i_* inflation, we retained the analytical NEXI formulation for this methodological study. The ultra-high gradient performance of the Connectome 2.0 system allows for very short gradient durations (δ = 5 ms), substantially mitigating the violation of the Narrow Pulse Approximation compared with clinical systems. Furthermore, the analytical Jacobian of the standard NEXI model provides the necessary numerical stability for rigorous CRLB benchmarking, avoiding the computational complexity of numerical solvers. Consequently, given the hardware capabilities and our focus on robust protocol comparison, standard NEXI provides a stable and sufficient approximation.

### 4.5 Denoising tradeoffs and noise model mismatch

Another key finding relates to the influence of signal-domain processing. Despite theoretical optimality, complex-valued MP-PCA denoising with Gaussian noise assumptions resulted in poorer model fits at high b-values than magnitude-domain denoising followed by Rician bias correction (Supplementary Fig. S4), a result also seen in Uhl et al. (2024c). This may reflect residual violations of noise model assumptions, especially in regions with low SNR or spatially correlated noise, arising from different reconstruction steps such as coil combination and parallel imaging (Henriques et al., 2023).

Future studies may benefit from incorporating more advanced denoising strategies, such as NORDIC (Moeller et al., 2021) or denoising models with spatially varying noise priors (Henriques et al., 2023), tailored specifically for cortical data. The possibility of using real-valued reconstructions to suppress Rician floor effects while preserving SNR remains a promising avenue for enhancing estimation robustness.

### 4.6 Clinical translation and cross-platform generalizability

While the proposed eight-feature protocol performs robustly on Siemens Connectome 2.0, its direct deployment on most clinical MRI systems is limited by hardware constraints. For example, Siemens Prisma-class scanners (maximum 80 mT/m) require longer δ and Δ to reach high b-values, reducing sensitivity to short-time exchange processes and violating the NPA that underlies the NEXI model. However, the emergence of clinical-grade scanners with ultra-high gradient performance, such as the GE Magnus (300 mT/m, 750 T/m/s) or the Siemens Cima.X (200 mT/m, 200 T/m/s), presents new opportunities. These systems are capable of running protocols similar to the one we have optimized, demonstrating that our data-driven approach is broadly applicable and not limited to a single research scanner. Prior work has shown that on standard clinical scanners, the SMEX model, which integrates full gradient waveforms, yields more accurate estimates (Olesen et al., 2022; Uhl et al., 2025).

Nevertheless, the XGBoost-SHAP-RFE optimization framework introduced here can be readily adapted to generate optimized subprotocols under these constraints. By re-training the XGBoost regressors on synthetic signals generated with Prisma-specific pulse timings and noise characteristics, it is possible to derive context-appropriate protocols that preserve the strengths of our approach while ensuring model validity. One of our previous works already begun applying SHAP-based optimization for clinical NEXI acquisition (Uhl et al., 2023), and our new findings further validate the utility of this pipeline for rapid, interpretable protocol design.

### 4.7 Toward multi-model and population-specific optimization

Finally, we note that the SHAP-RFE approach is model agnostic and supports extensions beyond NEXI. As the field moves toward multi-compartment models that incorporate soma (e.g., SANDIX) (Olesen et al., 2022; Uhl et al., 2024a), structural disorder (Novikov et al., 2023), or relaxation (Lee et al., 2020), protocol optimization strategies must evolve accordingly. Our method provides a principled route for empirically selecting features that maximize sensitivity across competing models or prioritize specific biological hypotheses. For instance, this approach could inform the development of free-waveform protocols aiming to enhance sensitivity to both cortical exchange and restriction, complementing existing frameworks such as that of Chakwizira et al. (2023).

Furthermore, future work may incorporate priors from pathology-specific cohorts or link optimization targets to downstream biomarkers (e.g., myelin water fraction, cortical thinning, clinical scores). The interpretability and modularity of SHAP facilitate such integration, offering an avenue for personalized or disease-aware protocol design that bridges methodological rigor with translational relevance. Beyond this, our optimization framework could be extended to other acquisition dimensions. For instance, future work could aim to optimize the number of diffusion directions per (b, Δ) combination. This would require a different pipeline than the one used for feature elimination, as simply removing a direction adds noise to the corresponding data point, which could bias the importance ranking and lead to unstable results.

Taken together, our findings demonstrate that XGBoost-SHAP-RFE-based protocol optimization is a powerful and adaptable tool for accelerating diffusion MRI acquisitions while preserving biological specificity. By empirically identifying informative (*b*, Δ) pairs under realistic noise and model uncertainty, we provide a scalable framework for optimizing complex biophysical imaging protocols. This framework is generalizable to other scanner platforms, populations, and research questions, and our work represents a key first step toward its broader application. It contributes a key advancement toward the widespread deployment of exchange-sensitive microstructural imaging in research and clinical applications.

## 5 Conclusion

We present a data-driven protocol optimization framework for Neurite Exchange Imaging (NEXI), leveraging explainable machine learning (XGBoost-SHAP-RFE) to identify a compact eight-feature acquisition scheme that maintains estimation accuracy, spatial specificity, and repeatability. Applied on the Connectome 2.0 platform, this reduced protocol cuts scan time nearly by half compared with the full 15-feature reference, while matching the performance of a rigorous CRLB optimization and significantly outperforming expert heuristic strategies.

Our results demonstrate a striking convergence between data-driven feature selection and theoretical A-optimality, confirming that XAI can effectively identify the information geometry of biophysical models directly from simulated signals. Crucially, the optimized protocol avoids the pitfalls of intuitive designs: it eliminates the estimation bias observed with dense mid-range sampling and mitigates the noise instability inherent to extreme corner sampling. This balance is particularly vital for robustly estimating challenging parameters such as *t_ex_* and *f* in the presence of realistic noise.

While not directly portable to clinical systems due to gradient constraints, the proposed optimization method is model agnostic and can be retrained for clinical MRI scanner platforms using realistic simulations. This flexible framework represents a critical step toward clinically viable, exchange-sensitive dMRI protocols for probing cortical microstructure in vivo.

## Supplementary Material

Supplementary Material

## Data Availability

The data that support the findings of this study are publicly available on OpenNeuro (accession number ds008771) at https://openneuro.org/datasets/ds008771. Access is unrestricted under the CC0 1.0 Universal Public Domain Dedication. The code used in this study is available at https://github.com/Mic-map/graymatter_swissknife. All code for XGBoost-SHAP-RFE optimization is publicly available at https://github.com/QuentinUhl/XAI-dMRI-protocol-optimization.
