# Supplementary material for: Reduced NEXI protocol for the quantification of human gray matter microstructure on the Connectome 2.0 scanner
Source: Imaging Neurosci (Camb). 2026 Oct 9;4:IMAG.a.1380. doi: 10.1162/IMAG.a.1380 (PMC13657203; doi:10.1162/IMAG.a.1380)
Supplement: Supplementary file 1 [file IMAG.a.1380_supp.pdf]

Supplementary Materials

Reduced NEXI protocol for the quantification of human gray matter microstructure on the Connectome 2.0 scanner

Quentin Uhl[1], Tommaso Pavan[1], Julianna Gerold[2], Kwok-Shing Chan[2], Yohan Jun[2], Shohei Fujita[2], Aneri Bhatt[2], Yixin Ma[2], Qiaochu Wang[1], Hong-Hsi Lee[2], Susie Y. Huang[2], Berkin Bilgic[2,*], Ileana Jelescu[1,*]

[1] Department of Radiology, Lausanne University Hospital (CHUV) and University of Lausanne, Lausanne, Switzerland

[2] Department of Radiology, Massachusetts General Hospital, Athinoula A. Martinos Center for Biomedical Imaging, Boston, MA, USA

[*] Joint last authorship

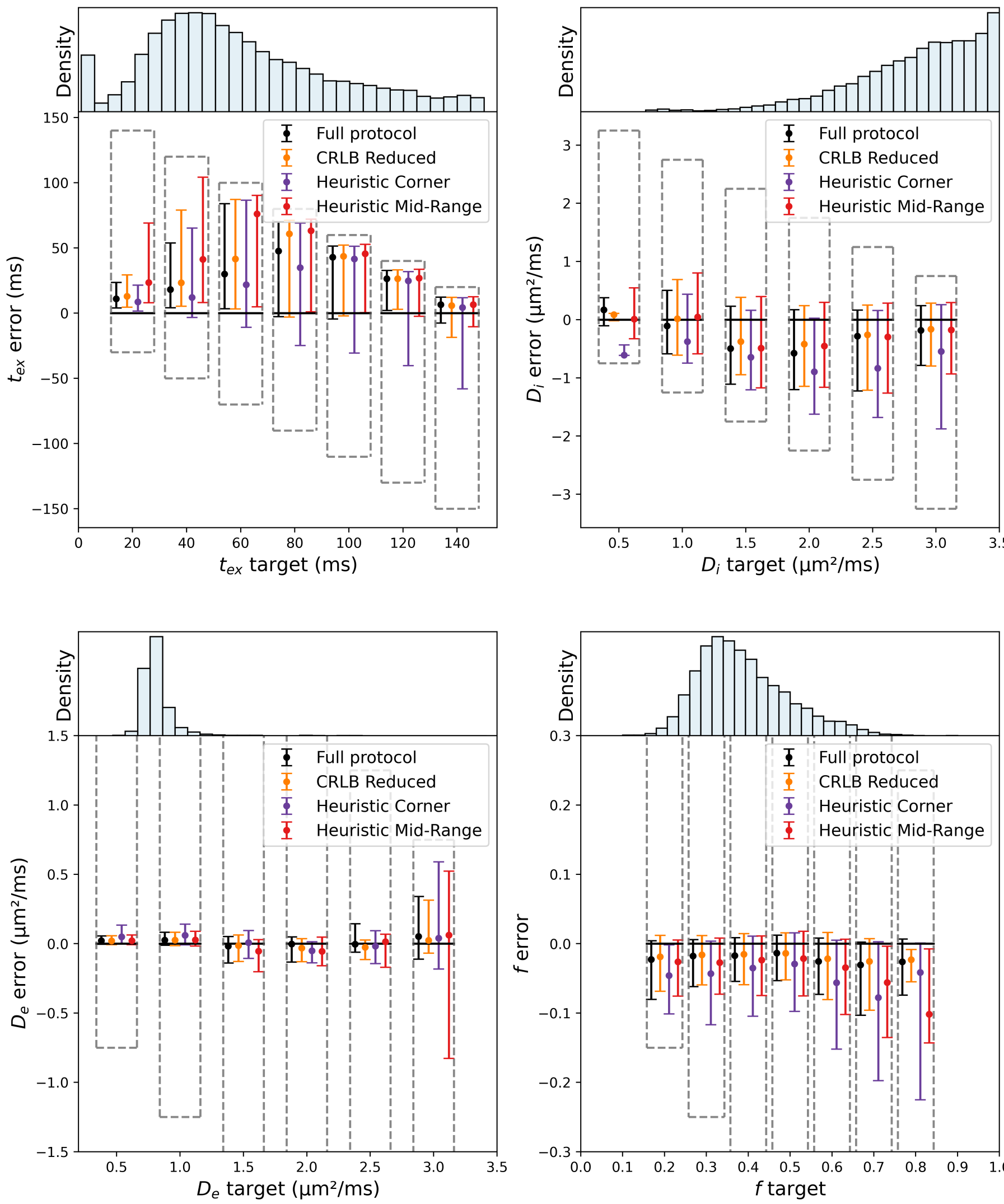

Figure S1. **Estimation errors from synthetic simulations: Full vs. CRLB and Heuristic subprotocols.** Boxplots show absolute errors in parameter estimation (t_{ex}, f, D_i, D_e) for synthetic NEXI signals corrupted with Gaussian noise (SNR=32±26). As in Figure 3, y-axis limits are scaled per parameter to improve visibility. Dashed boxes represent model fitting bounds. The Heuristic Mid-Range protocol (red) shows the largest errors for t_{ex}, confirming its poor sensitivity to exchange due to limited Δ sampling. The Heuristic Corner protocol (purple) exhibits increased variance for D_i, consistent with its in vivo instability. Both the CRLB (orange) and XAI (see Fig. 3) protocols maintain errors closer to the Full protocol baseline.

A.

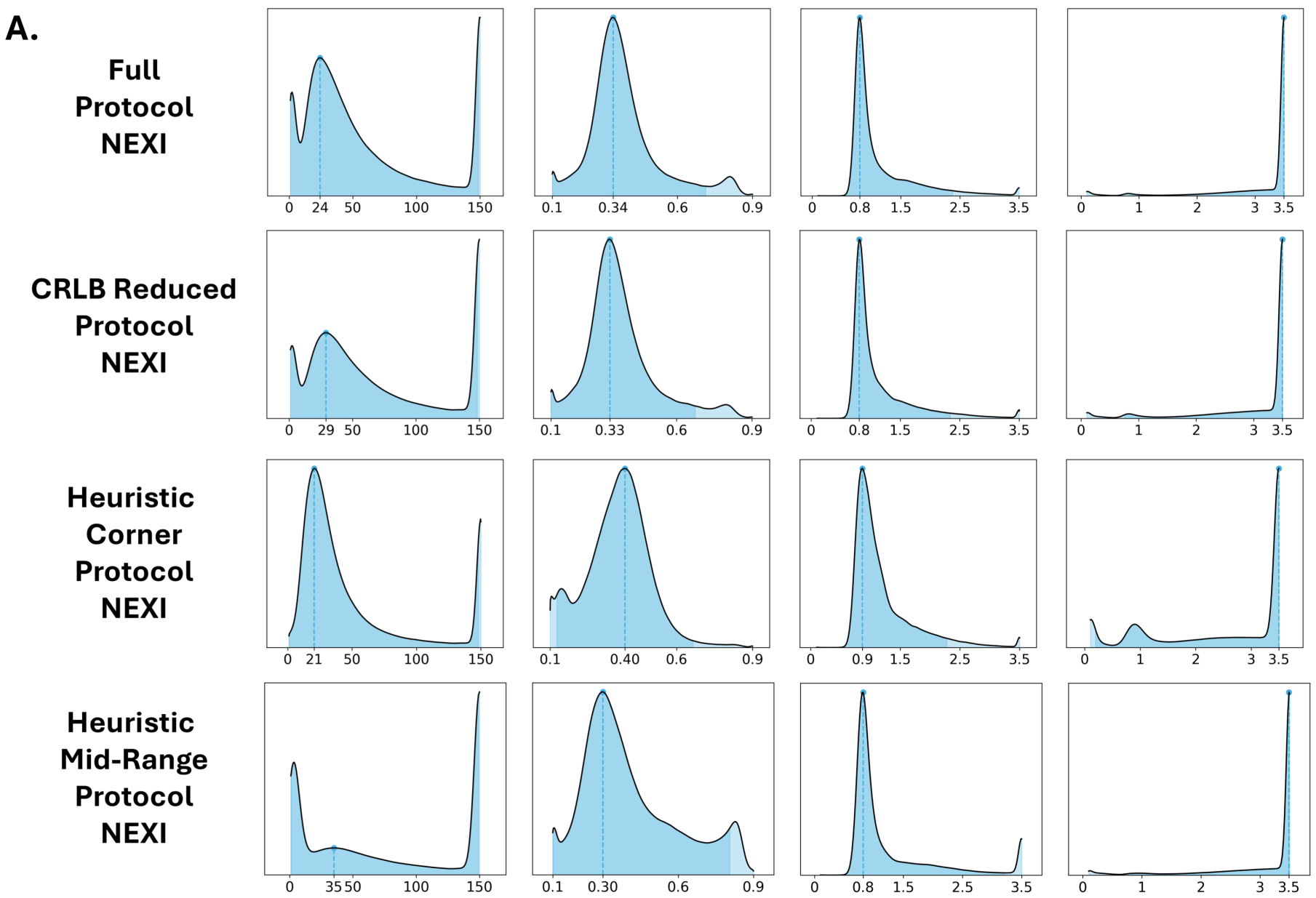

B.

DKT atlas ROI medians	t_{ex} (ms)	f	D_e (μm^2/ms)	D_i (μm^2/ms)
Full	**36** [1 - 130]	**0.36** [0.30 - 0.42]	**0.89** [0.68 - 1.09]	**3.49** [2.45 - 3.50]
CRLB Reduced	**44** [1 - 142]	**0.34** [0.29 - 0.40]	**0.89** [0.68 - 1.11]	**3.50** [2.21 - 3.50]
Heuristic Corner	**31** [1 - 74]	**0.38** [0.29 - 0.46]	**1.00** [0.78 - 1.22]	**3.48** [0.98 - 3.50]
Heuristic Mid-Range	**43** [1 - 147]	**0.33** [0.22 - 0.45]	**0.89** [0.66 - 1.11]	**3.50** [2.61 - 3.50]

Figure S2. **Distributions and summary of NEXI parameter estimates across DKT cortical regions for full, CRLB, and heuristic protocols.** (A) Kernel density estimates of voxel-level values for each NEXI parameter (t_{ex}, f, D_e, D_i), aggregated across all DKT ROIs and all subjects. The Full protocol (top row) serves as the reference, displaying characteristic unimodal distributions for f and D_e, and a right-skewed distribution for t_{ex}. The CRLB Reduced protocol (second row) closely mirrors the reference distributions, confirming its theoretical optimality. In contrast, the Heuristic Corner protocol (third row) exhibits a distorted distribution for D_i with a spurious secondary peak at low diffusivity values, reflecting the estimation instability caused by noise sensitivity at high b-values. The Heuristic Mid-Range protocol (bottom row) shows a sharpened and shifted distribution for t_{ex} toward the boundaries, indicative of the systematic bias caused by insufficient diffusion time sampling. The vertical dashed lines indicate the identified mode of each distribution. (B) Summary statistics of ROI-wise medians. While these aggregate metrics reveal specific biases, such as the inflated D_e for the Heuristic Corner protocol (1.00 vs 0.89 μm^2/ms), they largely mask the severe estimation instability observed in the distributions. Crucially, the median D_i for the Heuristic Corner protocol appears deceptively close to the reference, failing to reflect the substantial variance and distributional distortion visible in (A).

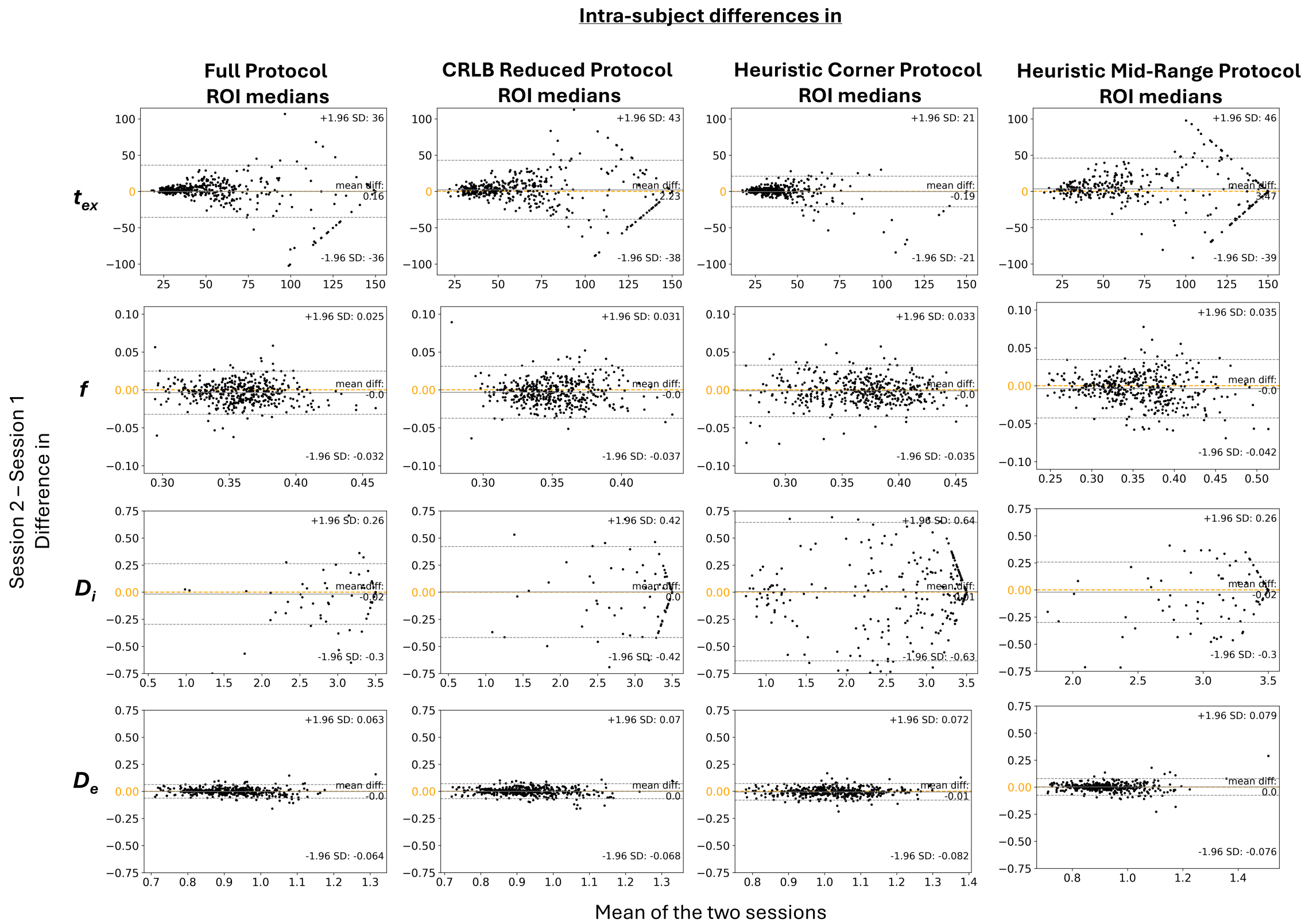

Figure S3. **Test–retest reproducibility of full, weighted-CRLB, and heuristic subprotocols.** Bland–Altman plots show intra-subject differences in DKT ROI medians between repeated sessions for t_{ex}, f, D_i, and D_e. Overall, all reduced protocols maintain a reproducibility generally comparable to the Full protocol (left) for stable parameters like f and D_e. However, critical differences emerge for exchange-sensitive metrics. Remarkably, the Heuristic Corner protocol (third column) demonstrates the best reproducibility for t_{ex}, with tighter limits of agreement (±21 ms) than even the Full protocol (±36 ms). This highlights the benefit of maximizing the geometric lever arm for constraining exchange parameters. However, this strategy fails for D_i, where noise sensitivity leads to marked instability (Limits: ±0.64 vs ±0.26 $\mu m^2/ms$ for Full). The Heuristic Mid-Range protocol (right) and CRLB Reduced protocol (second column) show balanced reproducibility profiles comparable to the Full protocol, without the instability observed in the Corner approach.

Supplementary Comparison Across Scanners and Pipelines

To contextualize our current results on the CONNECTOM 2.0 scanner, we compared group-level NEXI parameter estimates to those obtained in our previous studies using both CONNECTOM 1.0 and PRISMA scanners. The CONNECTOM 1.0 dataset comprised data from four healthy adults (Age: 30.5±3.8 years) acquired on a 3T scanner with 300 mT/m gradients. The acquisition protocol used b-values from 1.0 to 7.5 ms/µm^2, at four Δ from 20 to 49 ms, with a fixed gradient duration of δ=9 ms. The protocol totaled 700 volumes and 45 minutes of scan time Uhl et al. (2024c). The PRISMA dataset included data from 11 healthy adults (Age: 26.9±1.3 years) scanned on a 3T system with 80 mT/m gradients. This protocol, requiring a total of 27 minutes, sampled b-values from 1.0 to 5.0 ms/µm^2 at five Δ from 28.3 to 65.0 ms, with a fixed gradient duration of δ=16.5 ms and 20 directions per shell, totaling 325 volumes Uhl et al. (2025). The two supplementary tables below summarize values across cortical regions using either the mode of means (Table S1) or the mean of medians (Table S2), allowing fair comparison across acquisition hardware, denoising strategies, and modeling variants (e.g., standard NEXI vs. SMEX). Notably, t_{ex} is consistently longer and D_i reaches upper bounds more frequently in the CONNECTOM 2.0 data, likely reflecting its higher gradient strength and sensitivity to fast intra-neurite dynamics. At the same time, inter-scanner discrepancies in f and D_e remain modest. These cross-platform results highlight both the potential and challenges of interpreting NEXI parameters across hardware configurations and processing choices.

	t_{ex} (ms)	D_i ($\mu m^2/ms$)	D_e ($\mu m^2/ms$)	f
CONNECTOM 2.0 (Complex)	**55.7**	**3.26**	**1.12**	**0.37**
	[6.1 – 116.6]	[2.35 – 3.50]	[0.84 – 1.41]	[0.29 – 0.44]
PRISMA NEXI (Magnitude)	**41.1**	**2.61**	**1.59**	**0.51**
	[15.5 – 66.7]	[2.03 – 3.20]	[1.18 – 2.00]	[0.41 – 0.61]
PRISMA SMEX (Wide Pulses)	**36.8**	**2.45**	**1.23**	**0.42**
	[17.6 – 58.0]	[1.99 – 2.90]	[0.88 – 1.61]	[0.34 – 0.50]

Table S1. Summary of NEXI parameter estimates using the *mode of the means* across DKT cortical regions. This comparison includes data from CONNECTOM 2.0 (with complex-valued denoising), and PRISMA (magnitude-based denoising), processed using either standard NEXI or SMEX (a wide-pulse variant). Results show similar f and D_e values across systems, while t_{ex} is notably longer and D_i consistently higher in CONNECTOM 2.0, frequently reaching the upper model bound. These trends suggest enhanced sensitivity to fast exchange processes at shorter diffusion times, but also point toward possible model limitations.

	t_{ex} (ms)	D_i ($\mu m^2/ms$)	D_e ($\mu m^2/ms$)	f
CONNECTOM 1.0 (NEXI_{RM})	**42.3**	**3.35**	**0.92**	**0.38**
	[40.0 – 44.7]	[3.32 – 3.38]	[0.91 – 0.93]	[0.379 – 0.389]
CONNECTOM 2.0 (Complex)	**57.9**	**3.40**	**0.92**	**0.36**
	[23.2 – 150.0]	[2.26 – 3.50]	[0.75 – 1.12]	[0.30 – 0.41]

Table S2. Comparison of NEXI parameter estimates using the *mean of DKT medians* with 95% confidence intervals (CI).
Estimates are derived from two acquisition protocols: CONNECTOM 1.0 using magnitude denoising with the Rician mean model (NEXI_{RM}), and CONNECTOM 2.0 using complex-valued denoising. While f and D_e remain comparable between systems, t_{ex} is substantially higher in CONNECTOM 2.0. Additionally, D_i values in C2 reach the model's upper fitting bounds in many ROIs, potentially reflecting sensitivity to fast intra-neurite diffusion or the lack of explicit soma modeling.

Impact of denoising strategy on signal modeling and parameter estimation

Although complex-valued denoising is often favored in diffusion MRI for its minimal bias under Gaussian noise assumptions, its superiority for model fitting is not guaranteed in practice. In this section, we compare model–data agreement and parameter estimates between two processing pipelines: (i) complex-valued MP-PCA denoising with standard NEXI fitting, and (ii) magnitude denoising with Rician mean correction (NEXI_{RM}). To ensure statistical robustness, all models were fitted at the voxel level; the signals and curves shown hereafter represent the average of these individual voxel-wise fits across the cortical ribbon. Figure S4 evaluates model fit quality against mean cortical signals. This result emphasize the importance of empirically validating denoising choices, especially under conditions of high diffusion weighting or limited SNR, rather than relying solely on theoretical bias arguments.

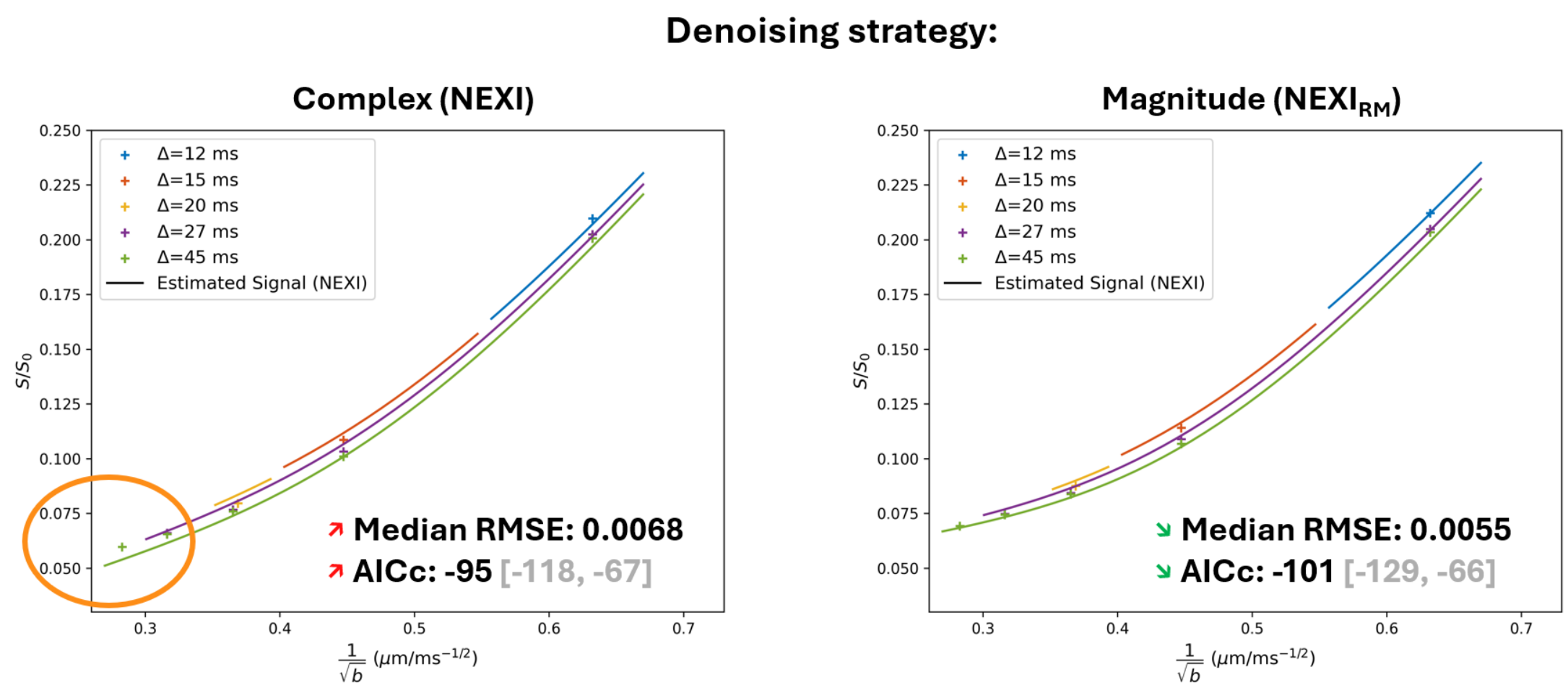

Figure S4. **Comparison of NEXI model fitting using complex vs. magnitude-denoised data.** Mean cortical diffusion signals are plotted as a function of $1/\sqrt{b}$ for each diffusion time Δ (color-coded), along with the corresponding NEXI model fit (black lines). Note that lower $1/\sqrt{b}$ values correspond to the highest diffusion weightings. Left: Complex-valued MP-PCA denoising followed by standard NEXI fitting. Right: Magnitude-based denoising followed by Rician-mean corrected NEXI fitting (NEXI_{RM}). Although complex denoising is theoretically less biased, it yields a higher residual median RMSE (0.0068) and worse AICc (-95) compared to the magnitude-based pipeline (RMSE $= 0.0055$, AICc $= -101$). Notably, a visible mismatch appears for high b-values (i.e., low $1/\sqrt{b}$) and long diffusion times ($\Delta = 45$ ms, green) in the complex case, suggesting a deviation between signal and model fit that is not present in the magnitude-based pipeline. This difference may reflect a residual model inadequacy at high diffusion weighting or subtle SNR-driven effects. These results underscore the need to empirically evaluate model–data agreement even under theoretically optimal denoising conditions.

Extension to High-Resolution Acquisition

Beyond designing a clinically viable protocol, we also assessed its performance in a high-resolution setting to address the challenge of Partial Volume Effects (PVE), which commonly confound microstructural measurements in cortical gray matter. One subject was scanned using the 8-feature optimized protocol at 1.6 mm isotropic resolution over a 60-minute session. The resulting NEXI parameter maps remained qualitatively consistent with those obtained at 2 mm resolution, as shown both on the cortical surface (Fig. S5) and in axial slices (Fig. S6). High-resolution acquisitions provided sharper delineation of the cortical ribbon and improved contrast at the gray-white matter boundary, particularly for f and D_i, with no indication of increased instability in model fitting. These results demonstrate that our reduced protocol can be leveraged for more accurate and reliable cortical mapping when extended scan durations are feasible.

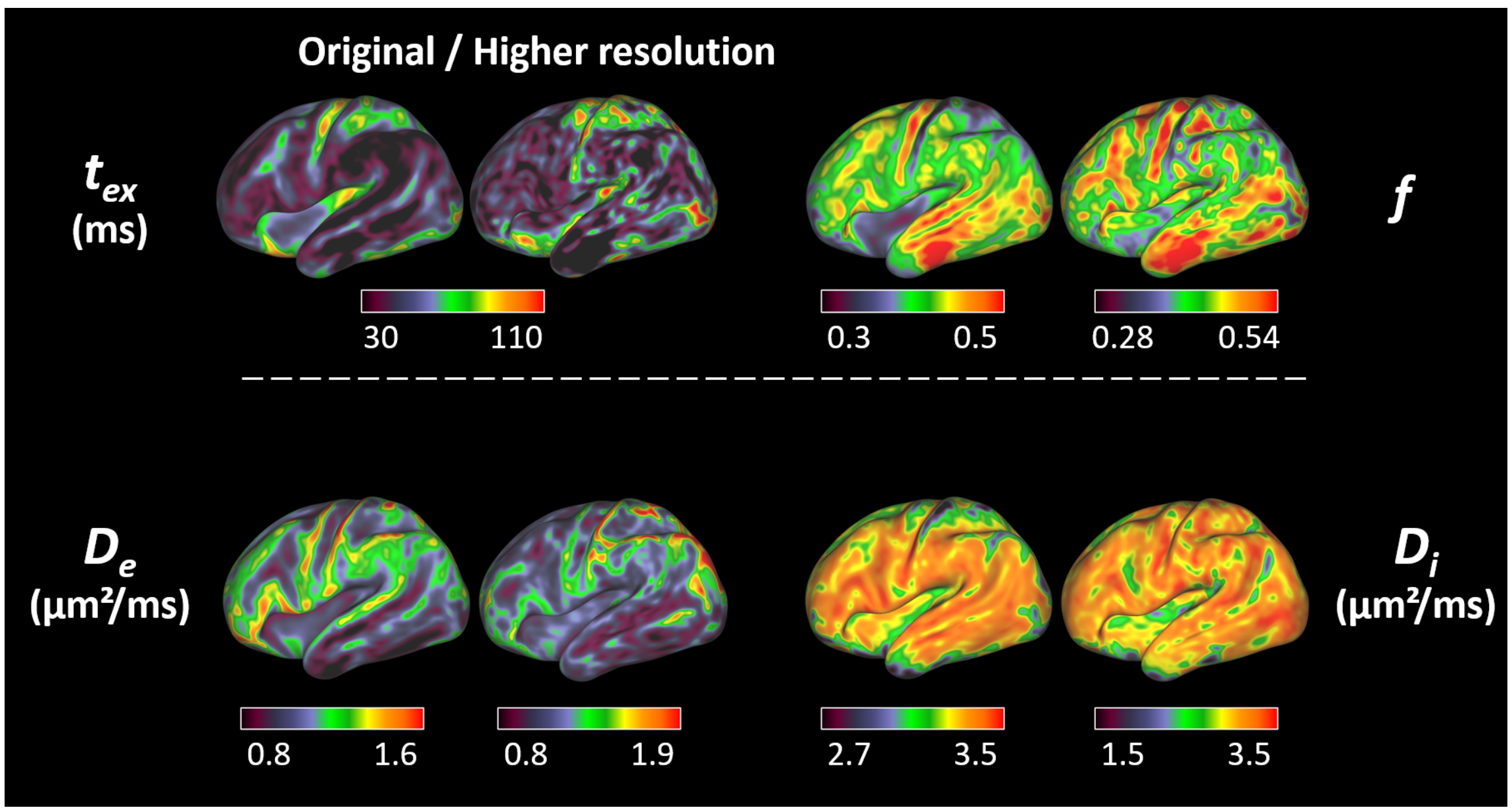

Figure S5. **Surface-based comparison of NEXI parameters at standard (2.0 mm) and high (1.6 mm) resolution.** Spatial distributions are largely preserved across resolutions, confirming the robustness of parameter estimation under increased spatial detail. Notably, subtle differences emerge in fine anatomical structures, particularly in f and t_{ex}, suggesting improved delineation of regional heterogeneity at higher resolution. Slight increases in parameter range (as seen in colorbars) may reflect reduced partial volume effects and enhanced anatomical specificity. This confirms that the reduced 8-feature protocol remains robust at higher spatial resolution and may support laminar or fine-grained microstructural analyses in longer acquisitions.

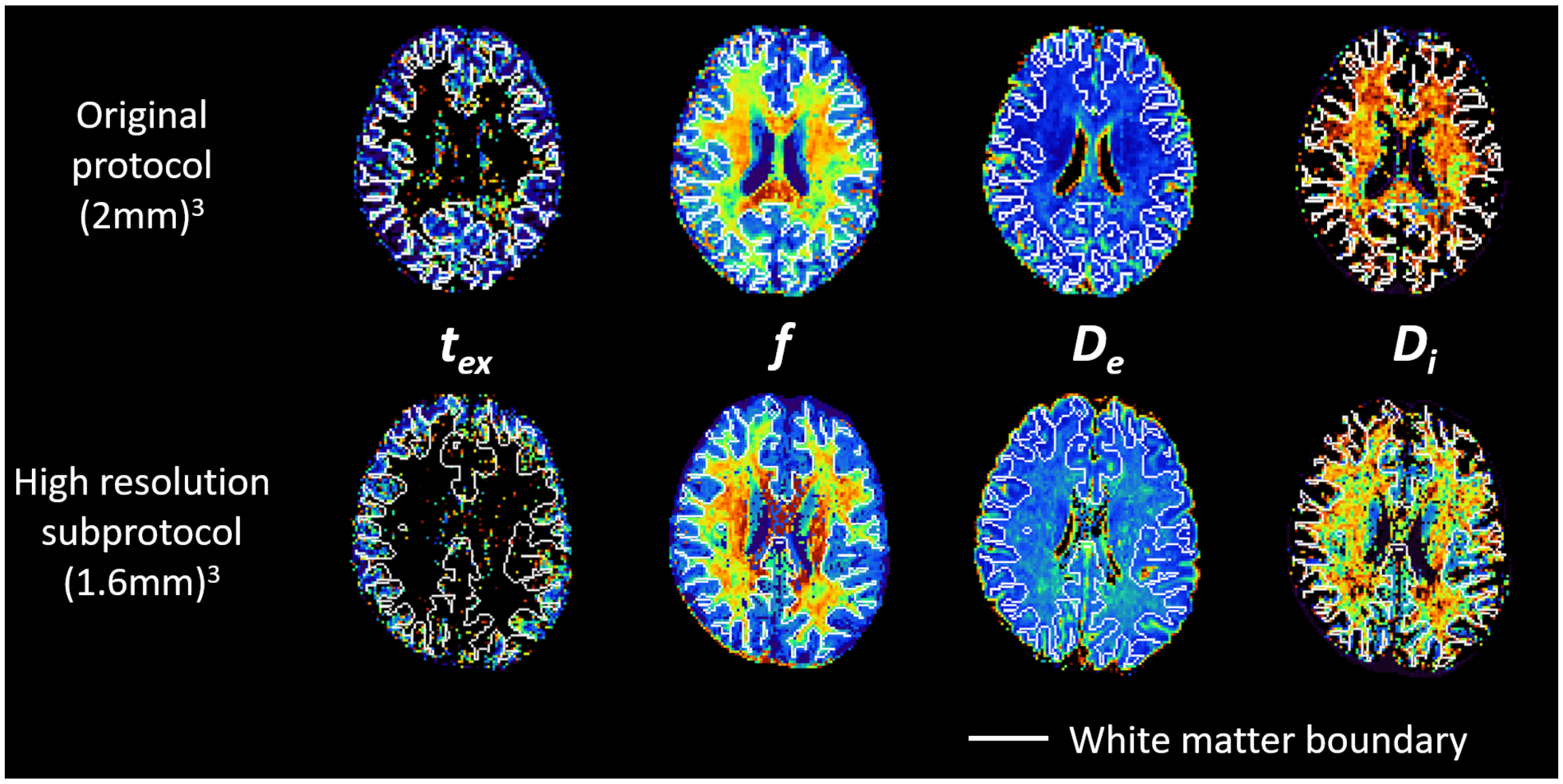

Figure S6. **Axial slice comparison of NEXI parameter maps at standard (2.0 mm) and high (1.6 mm) isotropic resolution.** Maps of t_{ex}, f, D_e, and D_i are displayed across the same mid-cortical axial slice in a single subject, comparing the original full protocol (top row) to the high-resolution version of the reduced 8-feature protocol (bottom row). Despite the smaller voxel size, parameter estimates remain qualitatively consistent, confirming the protocol's robustness across spatial resolutions. High-resolution maps reveal sharper gray–white matter boundaries (highlighted in white), improved delineation of cortical ribbon structure, and increased local contrast, especially for f and D_i. These enhancements suggest that the reduced protocol supports fine-grained anatomical mapping, and may be suitable for studies targeting laminar profiles or subtle cortical microstructural changes.

Convergence of Data-Driven and Theoretical Optimizations

In the main text, we demonstrated that the XAI-optimized protocol converges with the theoretical Weighted A-optimality (CRLB) criterion. To provide a comprehensive theoretical validation and ensure our results are not dependent on a single loss function, we also evaluated the Weighted D-optimality criterion based on the Fisher Information Matrix (FIM).

While A-optimality (used in the main text via the CRLB) minimizes the average relative variance, D-optimality seeks to minimize the volume of the confidence ellipsoid by maximizing the determinant of the information matrix. To ensure consistency with the weighted CRLB approach and account for the disparate units of NEXI parameters, we implemented a weighted FIM optimization defined as:

$$\max \det(\mathbf{J}^T \mathbf{J})$$

where $\mathbf{J}$ represents the weighted Jacobian, normalized by both the noise standard deviation associated with each acquisition and the ground-truth parameter values. This formulation aligns with the relative error minimization objective of the w-CRLB and XAI approaches.

As illustrated in Figure S7, the protocol resulting from this Weighted D-optimality optimization (Panel A) is strikingly similar to both the w-CRLB (Panel B) and XAI (Panel C) protocols presented in the main text. Specifically, the FIM-optimized protocol prioritizes the exact same "corners" of the sampling space (b_{min}/b_{max} and $\Delta_{min}/\Delta_{max}$) and shares 7 out of 8 features with the XAI protocol. This triple convergence across distinct optimization strategies, minimizing prediction error (XAI), minimizing parameter variance (CRLB), and maximizing information volume (FIM), strongly supports the global optimality of the selected 8-feature protocol.

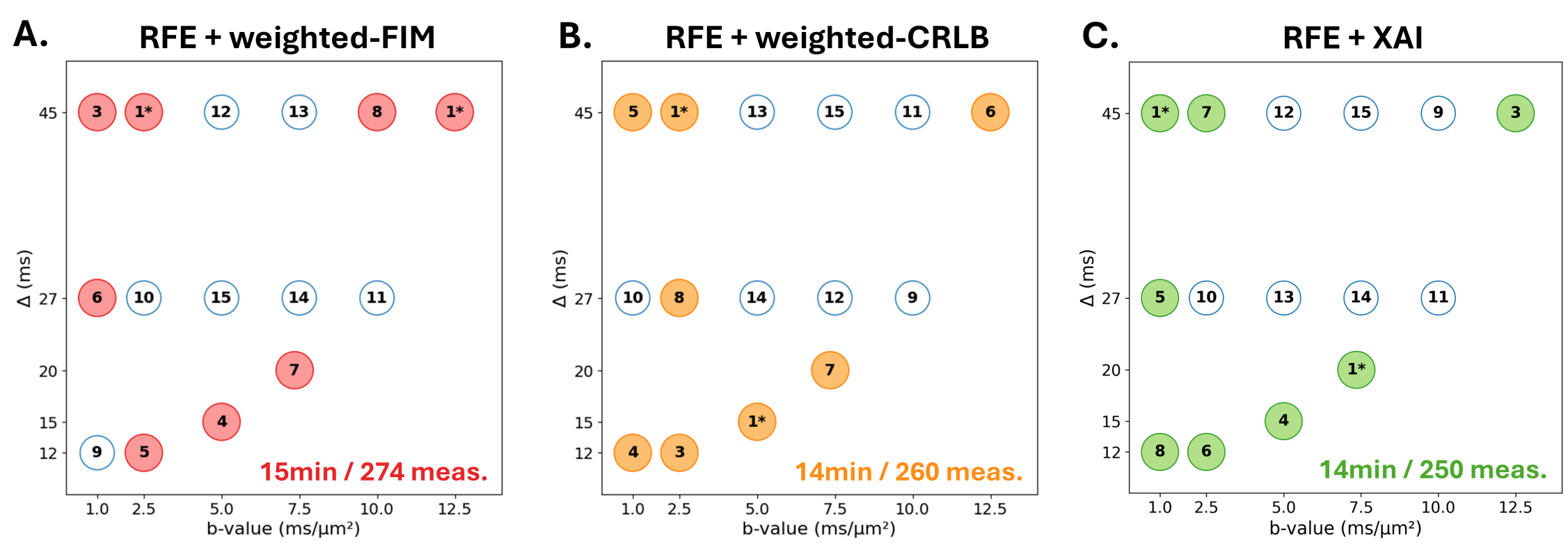

Figure S7. **Convergence of D-optimality (FIM) with A-optimality (CRLB) and XAI.** Comparison of the final feature sets selected by: **(A)** Weighted FIM RFE (D-optimality), **(B)** Weighted CRLB RFE (A-optimality, used in the main text), and **(C)** XAI-based RFE. Colored circles indicate the (b, Δ) combinations retained in the final 8-feature protocols, while empty circles represent features eliminated during the RFE process. The numbers inside the circles correspond to the shell indices of the full 15-feature protocol. All three methods independently converge towards a "corner-based" strategy, selecting the highest and lowest available b-values and diffusion times to maximize sensitivity. The FIM-optimized protocol confirms the trend observed with CRLB and XAI, validating that the chosen acquisition scheme is robust regardless of the specific theoretical optimality criterion applied.

Extended Optimization on a High-Density Continuous Grid

To demonstrate the robustness of our data-driven XAI pipeline and its capacity to scale beyond the initial 15-feature master protocol, we extended the optimization to a highly dense acquisition grid. A grid of 100 theoretical (b, Δ) combinations was generated. To ensure the physical viability of this search space, an exclusion mask was applied to strictly limit the maximum achievable b-value for any given Δ according to the hardware constraints of the Connectome 2.0 scanner ($G_{max} = 500$ mT/m, $\delta = 5$ ms). This filtering left 80 physically realizable (b, Δ) combinations. The number of diffusion directions for each synthetic shell was scaled following the heuristic rule $N_{dir} \propto \sqrt{b}$ (constrained between 20 and 64).

Both the XGBoost-SHAP-RFE pipeline and the weighted-CRLB optimization were executed on this dense 80-feature space. Figure S8 illustrates the final retained features. Remarkably, both the data-driven and theoretical optimizations exhibit a highly similar behavior: they predominantly select features at the extreme boundaries of the physically achievable parameter space, rather than clustering along the maximum gradient diagonal. Notably, the XAI approach maintains a slightly greater temporal diversity across Δ compared to the CRLB.

While both methods converge toward a common ”corner-heavy” protocol geometry, performing recursive elimination on such a dense grid introduces significant multicollinearity. Neighboring (b, Δ) points provide highly redundant information, making the early elimination steps delicate as many points share a very low individual impact. This observation provides strong post-hoc justification for our decision to restrict the primary in vivo optimization to a well-spaced 15-feature master protocol. By avoiding dense redundancy, the 15-feature approach stabilizes early feature ranking, ensures each point contributes unique information, and implicitly maintains compatibility with standard diffusion models, like DTI or DKI, by relying on conventional b-value shells.

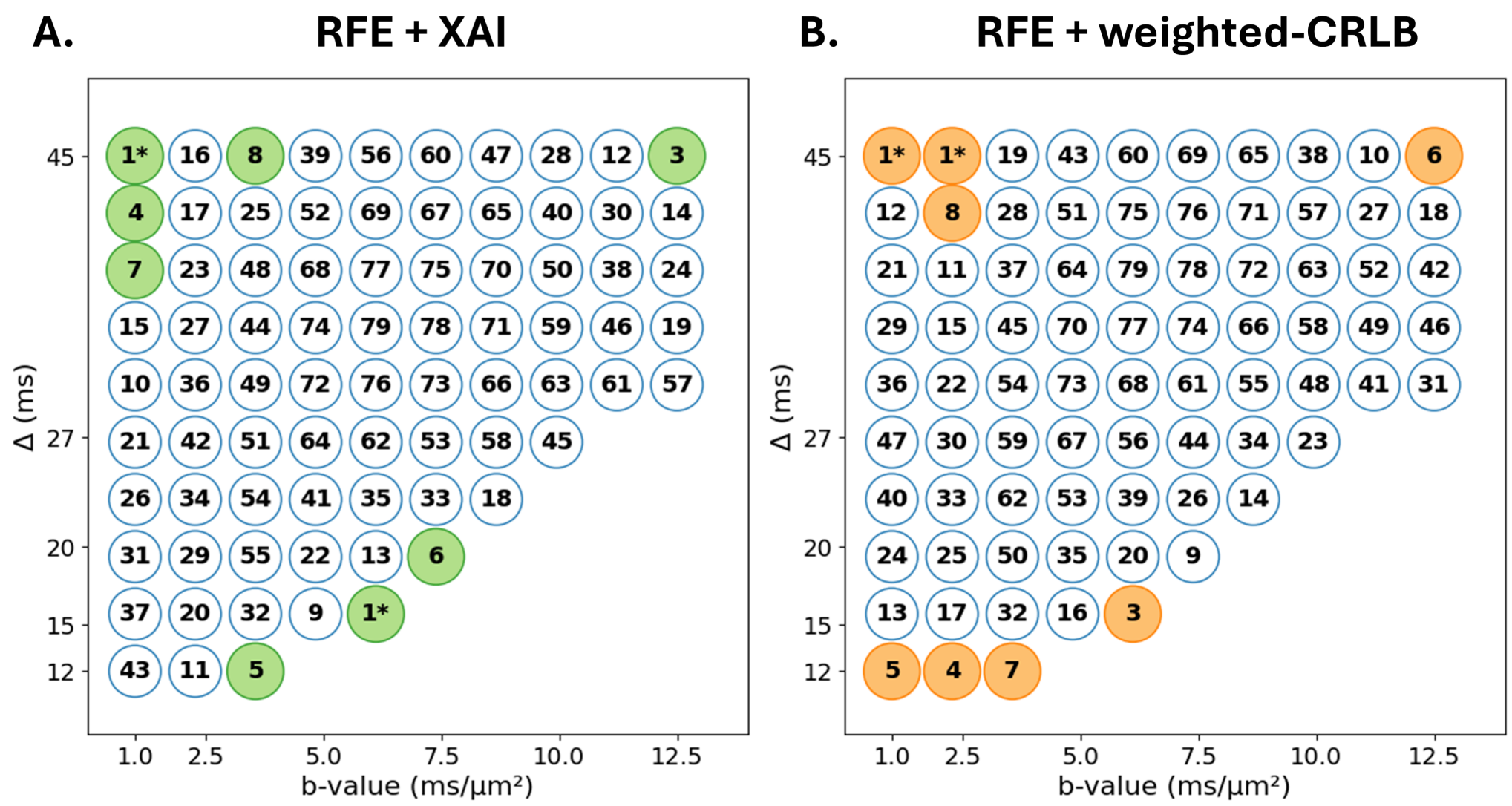

Figure S8. **Final optimized sub-protocols on a high-density, physically constrained grid.** XAI (left) and weighted-CRLB (right) optimizations were performed on an initial pool of 80 physically realizable (b, Δ) combinations. The numbers inside the circles indicate the elimination rank, with lower numbers representing the most important, last-remaining features. Both methods converge toward a similar macroscopic acquisition strategy, primarily retaining features (highlighted in green and orange) located at the extreme edges of the parameter space rather than along the maximum gradient efficiency diagonal. This confirms that the underlying information geometry relies heavily on boundary conditions, which is consistent with the subset selected from the 15-feature master protocol in the main text.
